# Drought and temporary migration in rural India: A comparative study across different socio-economic groups with a cross-sectional nationally representative dataset

**DOI:** 10.1371/journal.pone.0275449

**Published:** 2022-10-07

**Authors:** Badsha Sarkar, Swarup Dutta, Prashant Kumar Singh

**Affiliations:** 1 Department of Policy and Management Studies, TERI School of Advanced Studies, New Delhi, NCT of Delhi, India; 2 Division of Preventive Oncology & Population Health, National Institute of Cancer Prevention and Research, Indian Council of Medical Research, Noida, Uttar Pradesh, India; Neijiang Normal University, CHINA

## Abstract

Vast stretches of India comes under meteorological drought this year or the other. A huge population base in rural India are rendered highly vulnerable to this drought because of their primary dependency on agriculture and in turn they may respond through temporary migration out of the drought affected rural areas in search of alternative livelihoods. This study aims to investigate the association between drought and temporary migration in rural India by fitting binary logistic regression models on a cross-sectional dataset involving both National Sample Survey Organization (NSSO) 64^th^ round data and India Meteorological Department (IMD) rainfall data. The paper also examines whether this association varies across the different socio-economic groups. Out of the total temporary migrants generated in rural India in the study period, 99.46% migrated internally and 67.12% were rural to urban migrants. The study finds that there is a positive association between drought instances and probability of a household to have at least one temporary migrant member in rural India (OR 1.64 with p<0.001) while controlling all other covariates. The study also concludes that the probability of temporary migration on account of drought is more severe among the socio-economically marginalised sections of the rural population compared to their better-off counterparts.

## Introduction

India is a highly drought affected country. The percentage area under drought in the country has increased from 10% to 20% during the period 1959 to 2009 [1] along with an increase in intensity of monsoon droughts [2–4]. India is projected to face an increasing future trend of droughts which will be more severe, frequent, long and with greater spatial extent under warming climate scenarios [5, 6]. Characteristics such as high levels of poverty, high levels of borrowing, less crop diversity, agriculture as the major source of income and low level of agricultural insurance increase people’s vulnerability to drought [7]. Drought causes decrease in grain yields [8, 9]. One fifth of India’s population are living below international poverty line [10] and half of its population are still dependent on agriculture and these make the Indian population vulnerable to drought. Impacts of droughts can be felt across economy, environment and society. The most immediate socio-economic impacts of droughts are manifested in widespread disruption in rural societies on account of livelihood insecurity, greater indebtedness, reduction in the consumption of cereals, rise in school dropout rates, psychological and health problems, loss of social status among the most vulnerable sections, increase in social tensions and erosion of social capitals including outmigration of the population from the drought affected regions [11–13]. In the year 2020 alone, around 3,856,000 people were estimated to be internally displaced in India due to disasters including droughts [14].

The association between drought and human migration has been studied in many regions around the world including Brazil [15], Canada [16, 17], China [18], Ethiopia [19, 20], Kenya [21], Mali [22, 23], Nepal [24], Northern Latin America and the Caribbean [25], Syria [26], United States of America [27] etc. In India, a few studies have found a positive association between drought and migration by analysing Census of India and International Crops Research Institute for the Semi-Arid Tropics (ICRISAT) datasets [28–30]. This association between drought and migration is also evidenced in a few field based studies spread across different parts of India like Karnataka [31], Odisha [32–34], Tamil Nadu [35], West Bengal [36, 37], Hindu Kush-Himalayas [38] etc. This drought and migration nexus may vary across the different intersectional groups based on their different levels of access to resources and the different adaptation strategies adopted. A number of studies informs that the socio-economic backgrounds play an important role while taking decisions regarding migrations in the instances of drought or any other natural hazards in India [7, 32, 33, 35, 36, 39–42]. On this background, this study aims to investigate the association between drought and temporary migration in rural India by employing secondary datasets and applying quantitative methods. The paper also examines whether this association varies across the different socio-economic groups. After this short introduction, the paper will describe the datasets and statistical models used in the study, followed by a description of results and discussions and finally will end up in a conclusion.

## Materials and methods

### Socio-economic data

The socio-economic data source used for this study is from National Sample Survey Organisation (NSSO) 64^th^ round conducted between 1^st^ July 2007 and 30^th^ June 2008 called Employment and Unemployment and Migration Particulars with schedule 10.2 which is a nationally representative large sample survey. The survey was conducted under the aegis of National Sample Survey Office, Ministry of Statistics and Programme Implementation (MoSPI), Government of India (GoI). NSSO is in charge of carrying out extensive sample surveys across all over India. NSSO primarily collects data through national household surveys on numerous socioeconomic topics, Annual Survey of Industries (ASI) etc. In addition to conducting these surveys, NSSO gathers information on rural and urban pricing and contributes significantly to the advancement of crop statistics by supervising the area enumeration and crop estimating surveys conducted by state agencies. In addition, it keeps track of a frame of urban area units for use in sample surveys in cities. NSSO 64^th^ round is the latest available data on temporary migration from any government sources in India. The survey covered the whole of Indian Union except; Leh (Ladakh) and Kargil districts of Jammu and Kashmir, interior villages of Nagaland situated beyond 5km of the bus route and villages of Andaman and Nicobar Islands which remain inaccessible throughout the year. Data was collected from 12,589 first stage units (FSUs) - 7,921 rural and 4,668 urban—distributed over all the 35 states and union territories through a stratified multi-stage random sampling design. A sample of about 10 households was drawn from each selected village and urban block comprising a total 125,578 households (79,091 in rural areas and 46,487 in urban areas) and a total of 572,254 persons (374,294 in rural areas and 197,960 in urban areas). The data of this survey is available in public domain and can be accessed upon registration and request to ICSSR Data Service (available at http://www.icssrdataservice.in/). This source also contains more details about the sampling procedure of the survey. This study was approved by a Student Research Committee instituted by TERI School of Advanced Studies, New Delhi. Separate approval for conducting this research was not required since this study was based completely on secondary datasets generated by Government agencies and those datasets are freely available in the public domain.

### Sample size for the study

The study is cross-sectional in design and the unit of analysis is household. As discussed above the total sample size at household level in NSSO 64^th^ round is 125,578 which reduces to 79,091 after deducting the urban sample. After deducting the non-reporting cases of religion (2), social group (6), land possession (66), marital status (4), and educational status (19) the household sample size at rural areas further reduces to 78,994 which constitutes the total sample size for this study. The study classifies this total sample size into 3 equal economic groups (based on Monthly Per capita Consumption Expenditure [MPCE] tertiles) of lower economic group (LEG = 26,316), middle economic group (MEG = 26,342) and higher economic group (HEG = 26,336). The same total sample size is again decomposed into 4 social groups of Others (19,512), Other Backward Class (OBC = 30,974), Scheduled Castes (SC = 15,085) and Scheduled Tribes (ST = 13,423).

### Outcome variable

In this study a temporary migrant household is considered to be the dependent variable. To record data on temporary migration, NSSO 64^th^ round used the criteria of if a parson has stayed away from the place of enumeration for a continuous period of at least one month but less than six months during last one year for employment or in search of employment then that person is categorised as a temporary migrant. The present study codes a household with at least one temporary migrant member as 1 and a household without any temporary migrant member as 0. Out of the total temporary migrants in rural India, 99.46% migrated within national border and 67.12% were rural to urban migrants.

### Key explanatory variables

The meteorological data used in this study are from India Meteorological Department (IMD) gridded rainfall binary files (0.25*0.25) (available at https://www.imdpune.gov.in/Clim_Pred_LRF_New/Grided_Data_Download.html). Point level annual (1^st^ of January to 31^st^ of December) rainfall data were extracted with the help of GRADS 2.2 on yearly basis and were imported to QGIS 3.10.0 as shape files and interpolated (inverse distance weighted). All the data points falling within one district were averaged to get the annual rainfall data of that district. Districts which have experienced an annual rainfall negatively deviated by an amount of 40% or more than the normal rainfall (50 years average annual rainfall between 1951 to 2000 for that district [43]) for at least one year between five years preceding the NSSO survey i.e. between 2003 to 2007 [28, 44–46] are classified as drought (*DR*) affected. If the district had no drought year within the specified time period then the district was coded as 1 and a district with at least one drought year was coded as 2. The districts with a positive deviation of rainfall of at least 40% or more within the specified time period of 2003 to 2007 were classified as flood (*FL*) affected. Drought and flood data were merged with the NSSO data at district level.

### Covariates

Other than the meteorological variables, other background characteristics like demographic, economic, social and geographical, which may influence a household to undertake a temporary migration decision, have also been considered in this study either as household characteristics or as household head characteristics. The selection of the covariates depends on both the literature [47–50] and availability in NSSO 64^th^ round dataset. The study has considered eight household characteristics including household size (*SIZE*: up to 5 members and more than 5 members), whether the household has at least one non-resident (out-migrant) member (*OUT*), religion (*REL*: Hinduism, Islam and Others), social groups (*SOCIAL*: Others, Other Backward Class, Scheduled Castes and Scheduled Tribes), household land possession (*LAND*: up to 1 hectare, 1.01 to 4 hectares and more than 4 hectares), household occupation type (*OCCU*: self-employed in agriculture, self-employed in non-agriculture, agricultural labour, other labour and others), and MPCE categorised into 3 tertile classes with equal frequencies. The dimension of geographical variability was incorporated through the consideration of state of residence (*STATE*) with Bihar as the reference category. Four other variables have been employed to capture the characteristics of the household head as sex (*SEX*: male and female), age (*AGE*: 15–24, 25–34, 35–44, 45–54, 55–64, up to 14 & 64 and above), marital status (*MAR*: currently married, never married, widowed, divorced/separated), and educational level (*EDU*: not literate, literate but up to primary, above primary but up to secondary and above secondary). A more detail description of explanatory variables and their measurements are provided in S11 Table in S1 File. Pairwise correlation coefficient matrix shows that the r^2^ values range between +6 to -6 (S1-S8 Tables in S1 File).

### Model specifications

A univariate analysis for the description of the sample and a bivariate analysis for the distribution of temporary migration rates by different key and confounding variables are followed by 8 binary logistic regression models to assess the adjusted association between temporary migration and drought for the overall sample and each of the 3 economic and 4 social groups. Given the dichotomous nature of the dependent variable binary logistic regression seems to be more appropriate over an ordinary least square (OLS) regression [51]. The general logistic regression model for the probability of household *i* to have at least one temporary migrant member can be described through Eq 1;

$$P_{i}(MIGRA|1)=\frac{1}{1+e^{-z_{i}}}$$ Eq 1

$$\frac{P_{i}}{1-P_{i}}=e^{z_{i}}$$ Eq 2

$$In\;(\frac{P_{i}}{1-P_{i}})=z_{i}=\alpha +\beta X_{i}+\varepsilon_{i}$$ Eq 3

Eq 2 describes the odds ratio in the form of probability of household *i* to have at least one temporary migrant member divided by the probability complement. A logarithm of the odds ratio makes the logistic regression model a function of different independent variables (*X*_*i*_) in Eq 3 where *α* denotes intercept, *β* denotes coefficients and *ε_i_* denotes the error term. Eqs 4 and 5 depicts more comprehensive forms of the binary logistic regression models for the study.


$$In\;(\frac{P_{i}}{1-P_{i}})=\alpha +\beta_{1}{DR}_{i}+\beta_{2}{FL}_{i}+\beta_{3}{SIZE}_{i}+\beta_{4}{OUT}_{i}+\beta_{5}{REL}_{i}+\beta_{6}{SOCIAL}_{i}+\beta_{7}{LAND}_{i}+\beta_{8}{OCCU}_{i}+\beta_{9}{MPCE}_{i}+\beta_{10}{SEX}_{i}+\beta_{11}{AGE}_{i}+\beta_{12}{MAR}_{i}+\beta_{13}{EDU}_{i}+\beta_{14}{STATE}_{i}+\varepsilon_{i}$$ Eq 4

(Pi1−Pi)=e^(α+β1DRi+β2FLi+β3SIZEi+β4OUTi+β5RELi+β6SOCIALi+β7LANDi+β8OCCUi+β9MPCEi+β10SEXi+β11AGEi+β12MARi+β13EDUi+β14STATEi+εi) Eq 5

Data arrangement was done in SPSS 25 and Excel 13 and the regression models were built using STATA 15.

## Results

### Characteristics of the overall sample

Out of the total sample size of 78,994 households, 15.6% had at least one member who undertook a temporary migration during the last year (see Table 1 for unweighted sample characteristics and S12 Table in S1 File for weighted sample estimates). An amount of 9.3% and 13.2% households belong to a district which had faced a drought and flood for at least once in the last five years respectively. In terms of household characteristics, almost 70% of households are composed of up to 5 members. There are about 46.4% households where already at least one member of the household had migrated out (non-resident). In terms of religious affiliation, 78.6% households are affiliated with Hinduism. Almost 3/4^th^ of households belong to one or the other reserved social categories. About four out of five households (80.7%) possess only up to 1 hectare of land. In majority of the households (50.6%) the major source of income is self-employment either in agricultural or in non-agricultural activities whereas 33.8% households bring their major source of income from selling labour. There are about one third households with MPCE level below Rs. 774.20. Majority of the households (84.9%) are male headed and 50% of the household heads are of 35–54 years age group. As per personal details of household heads, 85.3% are currently married and 41.3% are not literate. In terms of geographical distribution of the sample, number of states contributing more than 5% share in migration are 6 which are Uttar Pradesh (11.4%), Bihar (8.9%), Andhra Pradesh (7.1%), West Bengal (6.9%), Maharashtra (6.4%) and Madhya Pradesh (5.6%).

**Table 1:** Characteristics of sample households in rural India for the study (unweighted), NSSO 64^th^ round (2007–2008) (in %).

Variables	Total sample (n = 78994)	LEG (n = 26316)	MEG (n = 26342)	HEG (n = 26336)	Others (n = 19512)	OBC (n = 30974)	SC (n = 15085)	ST (n = 13423)
**Dependent variable**								
**Temporary migrant**								
No	84.4	77.0	85.4	90.7	87.7	84.2	81.3	83.6
Yes	15.6	23.0	14.6	9.3	12.4	15.8	18.8	16.4
**Rainfall variable**								
**Drought**								
No	90.7	92.9	90.2	89.1	95.9	88.6	90.5	88.3
Yes	9.3	7.1	9.8	10.9	4.1	11.4	9.5	11.7
**Flood**								
No	86.8	86.0	87.1	87.2	89.5	84.9	88.5	85.2
Yes	13.2	14.0	12.9	12.8	10.6	15.1	11.5	14.8
**Household characteristics**								
**Household size**								
Up to 5	69.3	54.6	70.0	83.4	70.7	68.2	71.4	67.5
More than 5	30.7	45.4	30.0	16.6	29.3	31.8	28.6	32.5
**Household with out-migrant**								
Yes	46.4	39.4	45.5	54.2	51.4	47.0	44.7	39.5
No	53.6	60.6	54.5	45.8	48.6	53.0	55.3	60.5
**Religion**								
Hinduism	78.6	83.4	79.6	73.0	68.6	86.8	93.3	57.8
Islam	10.4	11.8	11.2	8.3	23.6	11.0	0.5	1.1
Others	11.0	4.8	9.2	18.8	7.8	2.2	6.2	41.1
**Social group**								
Others	24.7	16.3	23.4	34.4				
OBC	39.2	39.7	42.2	35.7				
SC	19.1	25.6	18.9	12.9				
ST	17.0	18.4	15.5	17.0				
**Land possession**								
Up to 1 hectare	80.7	85.8	80.9	75.5	76.2	80.9	92.7	73.3
1.01 to 4 hectares	17.3	13.2	17.4	21.1	20.2	16.9	7.0	25.3
More than 4 hectares	2.0	1.0	1.7	3.4	3.6	2.1	0.3	1.4
**Occupation type**								
Self-employed in agriculture	36.6	30.5	40.1	39.1	41.6	37.3	17.9	48.7
Self-employed in non-agriculture	14.0	12.6	14.7	14.8	16.1	16.5	12.8	6.6
Agricultural labour	22.7	36.6	22.0	9.4	12.6	21.6	39.3	21.0
Other labour	11.1	12.7	11.5	9.2	9.1	10.6	16.8	9.0
Others	15.6	7.7	11.7	27.5	20.7	14.0	13.3	14.7
**MPCE tertiles**								
LEG	33.3				22.0	33.7	44.6	36.2
MEG	33.4				31.6	35.9	32.9	30.5
HEG	33.3				46.4	30.4	22.5	33.4
**Household head characteristics**								
**Sex**								
Male	84.9	87.2	86.0	81.6	84.7	84.6	84.0	87.1
Female	15.1	12.8	14.0	18.4	15.3	15.4	16.0	12.9
**Age**								
15–24	2.5	2.2	2.3	3.0	2.2	2.5	3.1	2.4
25–34	16.3	19.2	16.3	13.5	14.0	16.0	18.9	17.8
35–44	24.2	27.8	23.6	21.3	22.9	24.3	24.8	25.5
45–54	25.9	24.1	26.3	27.5	26.1	25.3	25.1	28.0
55–64	18.6	16.2	19.0	20.6	19.5	19.3	16.9	17.7
Up to 14	0.2	0.3	0.1	0.2	0.1	0.1	0.2	0.6
65 and above	12.2	10.3	12.3	14.0	15.3	12.6	11.1	8.1
**Marital status**								
Currently married	85.3	87.8	86.3	81.8	85.4	85.3	85.0	85.4
Never married	2.2	1.1	1.5	4.0	2.4	2.0	1.8	2.6
Widowed	12.0	10.8	11.6	13.5	11.7	12.2	12.6	11.2
Divorced/separated	0.6	0.4	0.6	0.7	0.4	0.5	0.6	0.8
**Educational level**								
Not literate	41.3	54.6	42.9	26.4	29.1	42.7	54.6	40.8
Literate but up to primary	28.9	28.9	30.5	27.3	29.1	28.5	25.5	33.1
Above primary but up to secondary	22.1	14.1	21.6	30.7	29.0	21.8	15.6	20.0
Above secondary	7.7	2.5	5.1	15.7	12.8	7.0	4.3	6.1

LEG: Lower economic group; MEG: Middle economic group; HEG: Higher economic group; OBC: Other Backward Class; SC: Scheduled Castes; ST: Scheduled Tribes.

### Temporary migration rates by key explanatory variables and covariates across different socio-economic groups

Cross tabulation between migration and drought does not give any conclusive relationship between the two (see Table 2 for unweighted temporary migration rates and S13 Table in S1 File for weighted sample estimates). Households in the MEG, HEG and SC categories who belong to drought affected districts show marginally higher rates of temporary migration. On the other hand, the households in the overall sample, MEG, HEG, OBC and ST when belonging to flood affected districts have higher rates of temporary migration. Temporary migration rates are higher for households with family size more than 5 members and households with no out-migrant members across all the samples. Households affiliated with Islam have higher migration rates in all groups except HEG and ST. The SCs have higher migration rates in overall sample as well as in LEG and MEG. Households with up to 1 hectare of land possession have the higher migration rates except in HEG and ST. Households whose main income source is labour (either agricultural or non-agricultural) have higher migration rates compared to entrepreneurial households. Households within the lowest MPCE group produce the higher rates of migration. Male headed households produce more migrants over female headed households. Households with household heads aging between 45–54 have higher migration rates except MEG. Households with currently married household heads and household heads with no literacy produce more migrants. Among the larger states, Jharkhand is the highest migration rate generating state in the overall sample (26.9%), LEG (33.2%), MEG (24.9%), OBC (25.4%) and SC (38.5%) categories whereas Bihar produces the highest migration rate in the HEG (16.6%), West Bengal in Others (23.6%) and Gujarat in ST (29.2%) category.

**Table 2:** Temporary migration rates at household level by explanatory variables across different economic and social groups in rural India (unweighted), NSSO 64^th^ round (2007–2008) (in %).

Variables	Total sample	LEG	MEG	HEG	Others	OBC	SC	ST
**Rainfall variable**								
**Drought**								
No	15.7	23.1	14.5	9.1	12.4	16.0	18.7	16.6
Yes	15.3	21.0	16.1	10.9	11.8	14.6	19.3	15.1
*P-value*	*0*.*408*	*0*.*032*	*0*.*025*	*0*.*002*	*0*.*647*	*0*.*028*	*0*.*615*	*0*.*132*
**Flood**								
No	15.5	23.0	14.5	9.1	12.4	15.7	18.9	15.7
Yes	16.6	22.8	15.7	10.8	11.9	16.7	17.7	20.6
*P-value*	*0*.*003*	*0*.*764*	*0*.*051*	*0*.*001*	*0*.*470*	*0*.*101*	*0*.*220*	*<0*.*001*
**Household characteristics**								
**Household size**								
Up to 5	13.1	20.2	13.3	8.3	10.0	13.2	16.1	14.1
More than 5	21.4	26.4	17.8	14.1	18.0	21.5	25.5	21.3
*P-value*	*<0*.*001*	*<0*.*001*	*<0*.*001*	*<0*.*001*	*<0*.*001*	*<0*.*001*	*<0*.*001*	*<0*.*001*
**Household with out-migrant**								
Yes	10.4	17.2	9.5	6.2	8.3	9.8	12.8	12.9
No	20.2	26.8	18.9	13.0	16.7	21.2	23.6	18.8
*P-value*	*<0*.*001*	*<0*.*001*	*<0*.*001*	*<0*.*001*	*<0*.*001*	*<0*.*001*	*<0*.*001*	*<0*.*001*
**Religion**								
Hinduism	15.4	22.8	14.6	7.9	10.1	15.6	19.4	17.0
Islam	20.5	27.5	18.9	12.5	21.6	19.3	20.0	12.4
Others	12.5	16.0	9.4	13.2	4.7	9.6	8.5	15.7
*P-value*	*<0*.*001*	*<0*.*001*	*<0*.*001*	*<0*.*001*	*<0*.*001*	*<0*.*001*	*<0*.*001*	*0*.*061*
**Social group**								
Others	12.4	21.9	13.7	7.0				
OBC	15.8	23.1	15.3	8.4				
SC	18.8	25.2	16.1	9.8				
ST	16.4	20.7	12.5	15.5				
*P-value*	*<0*.*001*	*<0*.*001*	*<0*.*001*	*<0*.*001*				
**Land possession**								
Up to 1 hectare	16.2	23.5	15.3	9.0	13.2	16.6	18.9	16.0
1.01 to 4 hectares	13.8	20.5	12.4	10.8	10.2	13.4	16.4	17.7
More than 4 hectares	8.2	16.6	7.6	6.1	6.2	6.8	14.6	19.1
*P-value*	*<0*.*001*	*<0*.*001*	*<0*.*001*	*<0*.*001*	*<0*.*001*	*<0*.*001*	*0*.*098*	*0*.*037*
**Occupation type**								
Self-employed in agriculture	13.4	19.6	12.2	9.9	11.0	13.9	16.2	14.4
Self-employed in non-agriculture	16.1	24.0	15.8	9.6	12.9	17.0	18.7	16.1
Agricultural labour	20.9	25.0	17.5	12.6	19.0	21.4	20.7	21.7
Other labour	23.4	32.0	22.9	12.2	20.6	22.6	26.0	24.3
Others	7.3	10.2	7.9	6.2	7.0	5.9	7.3	11.0
*P-value*	*<0*.*001*	*<0*.*001*	*<0*.*001*	*<0*.*001*	*<0*.*001*	*<0*.*001*	*<0*.*001*	*<0*.*001*
**MPCE tertiles**								
LEG	23.0				21.9	23.1	25.2	20.7
MEG	14.6				13.7	15.3	16.1	12.5
UEG	9.3				7.0	8.4	9.8	15.5
*P-value*	*<0*.*001*				*<0*.*001*	*<0*.*001*	*<0*.*001*	*<0*.*001*
**Household head characteristics**								
**Sex**								
Male	17.1	24.7	15.8	10.4	13.5	17.4	20.7	17.6
Female	7.4	11.6	7.5	4.4	6.0	7.3	8.4	8.6
*P-value*	*<0*.*001*	*<0*.*001*	*<0*.*001*	*<0*.*001*	*<0*.*001*	*<0*.*001*	*<0*.*001*	*<0*.*001*
**Age**								
15–24	15.6	23.5	17.7	8.3	11.2	14.4	21.0	16.4
25–34	16.5	22.7	15.4	8.9	13.1	16.7	19.9	15.9
35–44	16.0	23.0	14.4	8.4	12.5	17.0	19.9	13.8
45–54	18.0	26.0	17.1	11.8	14.8	17.8	21.3	19.3
55–64	15.3	23.9	14.0	9.7	12.0	14.9	17.5	19.3
Up to 14	0.0	0.0	0.0	0.0	0.0	0.0	0.0	0.0
65 and above	9.8	15.6	9.1	6.0	8.2	10.5	10.1	11.0
*P-value*	*<0*.*001*	*<0*.*001*	*<0*.*001*	*<0*.*001*	*<0*.*001*	*<0*.*001*	*<0*.*001*	*<0*.*001*
**Marital status**								
Currently married	16.3	23.6	15.1	9.8	13.0	16.5	19.5	17.2
Never married	10.2	16.7	14.3	7.0	8.0	10.2	16.2	8.7
Widowed	12.3	19.3	11.4	7.4	9.1	12.8	14.8	12.7
Divorced/separated	8.8	11.0	12.9	4.2	7.0	8.6	10.8	8.9
*P-value*	*<0*.*001*	*<0*.*001*	*<0*.*001*	*<0*.*001*	*<0*.*001*	*<0*.*001*	*<0*.*001*	*<0*.*001*
**Educational level**								
Not literate	17.9	23.9	15.4	9.6	14.7	17.9	19.6	18.7
Literate but up to primary	16.0	23.2	14.6	9.9	13.7	16.1	19.6	15.4
Above primary but up to secondary	13.0	20.6	13.4	9.3	10.1	13.6	16.0	15.0
Above secondary	9.8	15.2	14.0	7.6	9.2	9.0	12.5	12.0
*P-value*	*<0*.*001*	*<0*.*001*	*0*.*005*	*<0*.*001*	*<0*.*001*	*<0*.*001*	*<0*.*001*	*<0*.*001*

LEG: Lower economic group; MEG: Middle economic group; HEG: Higher economic group; OBC: Other Backward Class; SC: Scheduled Castes; ST: Scheduled Tribes; *P-values* are of the Chi^2^ test.

### Associations between household characteristics, household head characteristics, geographical variables and temporary migration across the different socioeconomic groups

Households with more than 5 members and households without any out-migrant member are associated with higher probability for migration in comparison to their respective counterparts across all the groups after controlling all the covariates (Tables 3 and 4). Households affiliated with Islam are more probable to migration in comparison to Hindus across all the samples except OBC (OR 1.05 with p = 0.323) and SC (OR 0.98 with p = 0.933) while controlling all the covariates. In the overall sample and across all the three economic groups, the socially marginalised households are more probable to have at least one migrant member when compared to socially better off sections except MEG. With the increase in household land possession size the probability of migration goes on decreasing. In comparison to households where the main income source is self-employment in agriculture, the households with self-employment in non-agriculture, agricultural labour and other labour have higher probability for migration. The likelihood of migration decreases with increasing MPCE tertiles. The probability for member(s) of female headed household to undertake migration as compared to male headed households is invariably low (at a p-value less than 1%) in all the categories with the highest being observed among the STs (OR 0.63 with p<1%). Households with heads aged between 45–54 years show higher probability for migration in overall sample (OR 1.15 with p<10%), HEG (OR 1.39 with p<5%) and Others (OR 1.51 with p<5%). In terms of marital status of household heads, widowed headed households have higher probabilities for migration compared to households headed by currently married persons except STs. Households with illiterate household heads have higher probabilities for migration compared to others. Across all the states the households belonging to Bihar show higher probability of migration across all the groups except for overall, HEG and ST. ST households belonging to Nagaland (OR 3.63 with p<1%), Jharkhand (OR 1.75 with p<5%), Assam (OR 1.72 with p<10%) and Gujarat (OR 1.68 with p<10%) are more likely to be mobile compared to Bihar. Nagaland also scores higher probability in the overall sample (OR 2.24 with p<1%) and the HEG (OR 2.11 with p<1%).

**Table 3:** Odds ratios and associated significance levels from the binary logistic regression models assessing the association between explanatory variables and temporary migration across several economic groups in rural India, NSSO 64^th^ round (2007–2008).

Explanatory variables	Total sample		LEG		MEG		HEG	
	OR	*P>z*	OR	*P>z*	OR	*P>z*	OR	*P>z*
**Rainfall variable**								
**Drought**								
No ®								
Yes	1.64	*<0*.*001*	2.32	<0.001	1.55	<0.001	1.16	0.199
**Flood**								
No ®								
Yes	1.01	*0*.*693*	1.04	*0*.*417*	0.95	*0*.*407*	1.09	*0*.*271*
**Household characteristics**								
**Household size**								
Up to 5 ®								
More than 5	1.46	*<0*.*001*	1.34	*<0*.*001*	1.58	*<0*.*001*	1.65	*<0*.*001*
**Household with out-migrant**								
Yes ®								
No	1.80	*<0*.*001*	1.61	*<0*.*001*	2.01	*<0*.*001*	1.97	*<0*.*001*
**Religion**								
Hinduism ®								
Islam	1.15	*<0*.*001*	1.12	*0*.*044*	1.12	*0*.*074*	1.34	*<0*.*001*
Others	1.01	*0*.*919*	1.10	*0*.*329*	0.92	*0*.*483*	1.04	*0*.*745*
**Social group**								
Others ®								
OBC	1.10	*0*.*003*	1.15	*0*.*010*	1.02	*0*.*683*	1.07	*0*.*304*
SC	1.14	*<0*.*001*	1.20	*0*.*002*	1.03	*0*.*660*	1.21	*0*.*020*
ST	1.19	*<0*.*001*	1.26	*<0*.*001*	1.02	*0*.*791*	1.24	*0*.*027*
**Land possession**								
Up to 1 hectare ®								
1.01 to 4 hectares	1.03	*0*.*327*	1.11	*0*.*059*	0.94	*0*.*257*	0.97	*0*.*639*
More than 4 hectares	0.79	*0*.*018*	0.97	*0*.*860*	0.63	*0*.*014*	0.75	*0*.*063*
**Occupation type**								
Self-employed in agriculture ®								
Self-employed in non-agriculture	1.19	*<0*.*001*	1.17	*0*.*005*	1.25	*<0*.*001*	1.09	*0*.*211*
Agricultural labour	1.49	*<0*.*001*	1.38	*<0*.*001*	1.61	*<0*.*001*	1.66	*<0*.*001*
Other labour	2.09	*<0*.*001*	2.11	*<0*.*001*	2.36	*<0*.*001*	1.70	*<0*.*001*
Others	0.83	*<0*.*001*	0.72	*<0*.*001*	0.85	*0*.*050*	0.85	*0*.*019*
**MPCE tertiles**								
LEG ®								
MEG	0.76	*<0*.*001*						
HEG	0.62	*<0*.*001*						
**Household head characteristics**								
**Sex**								
Male ®								
Female	0.47	*<0*.*001*	0.43	*<0*.*001*	0.50	*<0*.*001*	0.52	*<0*.*001*
**Age**								
15–24 ®								
25–34	0.89	*0*.*112*	0.91	*0*.*382*	0.84	*0*.*171*	0.95	*0*.*745*
35–44	0.81	*0*.*003*	0.84	*0*.*133*	0.76	*0*.*029*	0.81	*0*.*186*
45–54	1.15	*0*.*061*	1.10	*0*.*405*	1.12	*0*.*360*	1.39	*0*.*032*
55–64	1.01	*0*.*929*	1.03	*0*.*783*	0.96	*0*.*751*	1.13	*0*.*432*
Up to 14	1.00		1.00		1.00		1.00	
65 and above	0.66	*<0*.*001*	0.64	*<0*.*001*	0.64	*0*.*001*	0.80	*0*.*187*
**Marital status**								
Currently married ®								
Never married	0.85	*0*.*072*	1.01	*0*.*954*	0.93	*0*.*659*	0.78	*0*.*083*
Widowed	1.32	*<0*.*001*	1.41	*<0*.*001*	1.22	*0*.*013*	1.29	*0*.*005*
Divorced/separated	0.89	*0*.*493*	0.78	*0*.*464*	1.27	*0*.*340*	0.57	*0*.*135*
**Educational level**								
Not literate ®								
Literate but up to primary	0.96	*0*.*161*	1.00	*0*.*978*	0.92	*0*.*073*	1.00	*0*.*947*
Above primary but up to secondary	0.85	*<0*.*001*	0.83	*<0*.*001*	0.85	*0*.*002*	0.89	*0*.*092*
Above secondary	0.79	*<0*.*001*	0.59	*<0*.*001*	0.94	*0*.*468*	0.83	*0*.*029*
**Geographical variable**								
**State**								
Bihar ®								
Jammu and Kashmir	0.81	*0*.*016*	0.54	*0*.*012*	0.66	*0*.*002*	0.83	*0*.*242*
Himachal Pradesh	0.30	*<0*.*001*	0.40	*<0*.*001*	0.28	*<0*.*001*	0.26	*<0*.*001*
Punjab	0.14	*<0*.*001*	0.12	*<0*.*001*	0.06	*<0*.*001*	0.19	*<0*.*001*
Chandigarh								
Uttaranchal	0.28	*<0*.*001*	0.24	*<0*.*001*	0.19	*<0*.*001*	0.39	*<0*.*001*
Haryana	0.27	*<0*.*001*	0.20	*<0*.*001*	0.22	*<0*.*001*	0.32	*<0*.*001*
Delhi	0.05	*<0*.*001*					0.06	*<0*.*001*
Rajasthan	0.50	*<0*.*001*	0.46	*<0*.*001*	0.51	*<0*.*001*	0.50	*<0*.*001*
Uttar Pradesh	0.83	*<0*.*001*	0.85	*0*.*006*	0.79	*0*.*001*	0.66	*0*.*001*
Sikkim	0.08	*<0*.*001*	0.04	*<0*.*001*	0.06	*<0*.*001*	0.13	*<0*.*001*
Arunachal Pradesh	0.57	*<0*.*001*	0.29	*<0*.*001*	0.53	*0*.*004*	0.92	*0*.*651*
Nagaland	2.24	*<0*.*001*	0.94	*0*.*942*	1.30	*0*.*341*	2.11	*<0*.*001*
Manipur	0.08	*<0*.*001*	0.09	*<0*.*001*	0.06	*<0*.*001*	0.11	*<0*.*001*
Mizoram	0.18	*<0*.*001*			0.25	*<0*.*001*	0.17	*<0*.*001*
Tripura	0.46	*<0*.*001*	0.28	*<0*.*001*	0.44	*<0*.*001*	0.69	*0*.*013*
Meghalaya	0.35	*<0*.*001*	0.09	*<0*.*001*	0.38	*<0*.*001*	0.49	*<0*.*001*
Assam	0.63	*<0*.*001*	0.49	*<0*.*001*	0.63	*<0*.*001*	0.69	*0*.*011*
West Bengal	0.88	*0*.*007*	1.06	*0*.*383*	0.70	*<0*.*001*	0.63	*0*.*001*
Jharkhand	0.94	*0*.*277*	0.97	*0*.*742*	0.91	*0*.*381*	0.64	*0*.*025*
Orissa	0.40	*<0*.*001*	0.47	*<0*.*001*	0.29	*<0*.*001*	0.12	*<0*.*001*
Chhattisgarh	0.41	*<0*.*001*	0.43	*<0*.*001*	0.36	*<0*.*001*	0.38	*0*.*001*
Madhya Pradesh	0.53	*<0*.*001*	0.49	*<0*.*001*	0.57	*<0*.*001*	0.48	*<0*.*001*
Gujarat	0.62	*<0*.*001*	0.60	*<0*.*001*	0.66	*<0*.*001*	0.54	*<0*.*001*
Daman and Diu	0.21	*<0*.*001*			0.15	*0*.*074*	0.23	*<0*.*001*
Dadra and Nagar Haveli	0.02	*<0*.*001*			0.04	*0*.*001*		
Maharashtra	0.34	*<0*.*001*	0.46	*<0*.*001*	0.25	*<0*.*001*	0.26	*<0*.*001*
Andhra Pradesh	0.43	*<0*.*001*	0.59	*<0*.*001*	0.36	*<0*.*001*	0.24	*<0*.*001*
Karnataka	0.64	*<0*.*001*	0.80	*0*.*007*	0.47	*<0*.*001*	0.56	*<0*.*001*
Goa	0.24	*<0*.*001*	0.14	*0*.*065*	0.21	*0*.*003*	0.29	*0*.*020*
Lakshadweep	0.11	*<0*.*001*			0.13	*0*.*053*	0.11	*0*.*003*
Kerala	0.23	*<0*.*001*	0.15	*<0*.*001*	0.20	*<0*.*001*	0.29	*<0*.*001*
Tamil Nadu	0.31	*<0*.*001*	0.15	*<0*.*001*	0.36	*<0*.*001*	0.61	*0*.*005*
Pondicherry	0.36	*<0*.*001*	0.03	*0*.*001*	0.24	*0*.*002*	0.92	*0*.*800*
Andaman and Nicobar	0.10	*<0*.*001*	0.22	*0*.*155*	0.03	*0*.*001*	0.13	*<0*.*001*
**Constant**	0.21	*<0*.*001*	0.23	*<0*.*001*	0.18	*<0*.*001*	0.11	*<0*.*001*
**Number of obs**	78,754		26,155		26,282		26,142	
**LR chi2(62)**	7458.70		1998.62		2086.92		1916.29	
**Prob > chi2**	0.00		0.00		0.00		0.00	
**Pseudo R2**	0.11		0.07		0.10		0.12	
**Log likelihood**	-30482.85		-13146.84		-9911.84		-7171.28	

LEG: Lower economic group; MEG: Middle economic group; HEG: Higher economic group; OBC: Other Backward Class; SC: Scheduled Castes; ST: Scheduled Tribes; OR denotes odds ratio; ® denotes the reference category.

**Table 4:** Odds ratios and associated significance levels from the binary logistic regression models assessing the association between explanatory variables and temporary migration across several social groups in rural India, NSSO 64^th^ round (2007–2008).

Explanatory variables	Others			OBC		SC		ST	
	OR	*P>z*		OR	*P>z*	OR	*P>z*	OR	*P>z*
**Rainfall variable**									
**Drought**									
No ®									
Yes	1.08	*0*.*620*		1.95	*<0*.*001*	1.92	*<0*.*001*	1.59	*<0*.*001*
**Flood**									
No ®									
Yes	0.91	*0*.*324*		0.93	*0*.*212*	0.86	*0*.*081*	1.74	*<0*.*001*
**Household characteristics**									
**Household size**									
Up to 5 ®									
More than 5	1.55	*<0*.*001*		1.38	*<0*.*001*	1.45	*<0*.*001*	1.53	*<0*.*001*
**Household with out-migrant**									
Yes ®									
No	1.88	*<0*.*001*		2.00	*<0*.*001*	1.74	*<0*.*001*	1.44	*<0*.*001*
**Religion**									
Hinduism ®									
Islam	1.25	*<0*.*001*		1.05	*0*.*323*	0.98	*0*.*933*	2.27	*0*.*020*
Others	0.71	*0*.*038*		1.26	*0*.*123*	1.06	*0*.*717*	1.20	*0*.*113*
**Land possession**									
Up to 1 hectare ®									
1.01 to 4 hectares	1.03	*0*.*721*		1.03	*0*.*610*	1.01	*0*.*949*	1.14	*0*.*049*
More than 4 hectares	0.91	*0*.*576*		0.65	*0*.*010*	0.97	*0*.*945*	1.22	*0*.*334*
**Occupation type**									
Self-employed in agriculture ®									
Self-employed in non-agriculture	0.94	*0*.*372*		1.22	*<0*.*001*	1.16	*0*.*083*	1.47	*<0*.*001*
Agricultural labour	1.32	*<0*.*001*		1.54	*<0*.*001*	1.26	*0*.*002*	1.88	*<0*.*001*
Other labour	1.93	*<0*.*001*		1.99	*<0*.*001*	1.92	*<0*.*001*	2.52	*<0*.*001*
Others	0.88	*0*.*117*		0.70	*<0*.*001*	0.77	*0*.*021*	1.04	*0*.*655*
**MPCE tertiles**									
LEG ®									
MEG	0.76	*<0*.*001*		0.79	*<0*.*001*	0.73	*<0*.*001*	0.71	*<0*.*001*
HEG	0.59	*<0*.*001*		0.62	*<0*.*001*	0.61	*<0*.*001*	0.74	*<0*.*001*
**Household head characteristics**									
**Sex**									
Male ®									
Female	0.51	*<0*.*001*		0.46	*<0*.*001*	0.39	*<0*.*001*	0.63	*<0*.*001*
**Age**									
15–24 ®									
25–34	1.12	*0*.*541*		0.95	*0*.*668*	0.75	*0*.*038*	0.85	*0*.*360*
35–44	1.00	*0*.*993*		0.90	*0*.*391*	0.70	*0*.*011*	0.68	*0*.*027*
45–54	1.51	*0*.*023*		1.20	*0*.*139*	0.96	*0*.*746*	1.15	*0*.*416*
55–64	1.32	*0*.*141*		1.04	*0*.*749*	0.80	*0*.*118*	1.10	*0*.*590*
Up to 14	1.00			1.00		1.00		1.00	
65 and above	0.92	*0*.*647*		0.75	*0*.*027*	0.45	*<0*.*001*	0.67	*0*.*048*
**Marital status**									
Currently married ®									
Never married	0.87	*0*.*450*		0.83	*0*.*221*	1.00	*0*.*995*	0.83	*0*.*387*
Widowed	1.21	*0*.*062*		1.38	*<0*.*001*	1.54	*<0*.*001*	1.02	*0*.*850*
Divorced/separated	0.87	*0*.*761*		0.82	*0*.*489*	0.89	*0*.*734*	0.85	*0*.*644*
**Educational level**									
Not literate ®									
Literate but up to primary	1.04	*0*.*568*		0.95	*0*.*195*	1.00	*0*.*966*	0.94	*0*.*296*
Above primary but up to secondary	0.89	*0*.*095*		0.85	*0*.*001*	0.82	*0*.*006*	0.81	*0*.*008*
Above secondary	0.95	*0*.*604*		0.73	*<0*.*001*	0.80	*0*.*095*	0.61	*0*.*001*
**Geographical variable**									
**State**									
Bihar ®									
Jammu and Kashmir	0.82	*0*.*108*		0.63	*0*.*012*	0.79	*0*.*342*	0.60	*0*.*519*
Himachal Pradesh	0.21	*<0*.*001*		0.47	*0*.*009*	0.41	*<0*.*001*	0.40	*0*.*019*
Punjab	0.23	*<0*.*001*		0.16	*<0*.*001*	0.11	*<0*.*001*		
Chandigarh									
Uttaranchal	0.32	*<0*.*001*		0.21	*<0*.*001*	0.28	*<0*.*001*		
Haryana	0.28	*<0*.*001*		0.21	*<0*.*001*	0.30	*<0*.*001*		
Delhi	1.00			0.43	*0*.*260*				
Rajasthan	0.48	*<0*.*001*		0.44	*<0*.*001*	0.65	*<0*.*001*	0.72	*0*.*243*
Uttar Pradesh	0.71	*0*.*002*		0.80	*<0*.*001*	0.88	*0*.*124*	1.36	*0*.*448*
Sikkim	0.03	*<0*.*001*		0.06	*<0*.*001*	0.05	*0*.*003*	0.24	*<0*.*001*
Arunachal Pradesh	1.12	*0*.*662*		0.43	*0*.*221*			0.94	*0*.*843*
Nagaland	1.66	*0*.*470*		0.38	*0*.*219*			3.63	*<0*.*001*
Manipur	0.26	*<0*.*001*		0.07	*<0*.*001*	0.16	*0*.*073*	0.10	*<0*.*001*
Mizoram						2.40	*0*.*541*	0.27	*<0*.*001*
Tripura	0.55	*<0*.*001*		0.68	*0*.*008*	0.48	*<0*.*001*	0.57	*0*.*054*
Meghalaya	1.00	*0*.*992*		0.25	*0*.*103*			0.50	*0*.*026*
Assam	0.52	*<0*.*001*		0.49	*<0*.*001*	0.59	*0*.*039*	1.72	*0*.*060*
West Bengal	0.86	*0*.*122*		0.83	*0*.*158*	0.88	*0*.*156*	1.35	*0*.*288*
Jharkhand	0.86	*0*.*454*		0.86	*0*.*089*	1.33	*0*.*037*	1.75	*0*.*045*
Orissa	0.25	*<0*.*001*		0.28	*<0*.*001*	0.48	*<0*.*001*	1.07	*0*.*803*
Chhattisgarh	0.71	*0*.*278*		0.49	*<0*.*001*	1.03	*0*.*870*	0.45	*0*.*006*
Madhya Pradesh	0.21	*<0*.*001*		0.46	*<0*.*001*	0.80	*0*.*038*	1.05	*0*.*843*
Gujarat	0.36	*<0*.*001*		0.54	*<0*.*001*	0.56	*0*.*003*	1.68	*0*.*060*
Daman and Diu				0.35	*0*.*011*			0.41	*0*.*254*
Dadra and Nagar Haveli								0.04	*0*.*003*
Maharashtra	0.21	*<0*.*001*		0.36	*<0*.*001*	0.43	*<0*.*001*	0.66	*0*.*132*
Andhra Pradesh	0.26	*<0*.*001*		0.40	*<0*.*001*	0.54	*<0*.*001*	1.20	*0*.*524*
Karnataka	0.59	*<0*.*001*		0.62	*<0*.*001*	0.79	*0*.*056*	0.83	*0*.*561*
Goa	0.19	*0*.*002*		0.29	*0*.*025*	0.31	*0*.*269*		
Lakshadweep								0.11	*0*.*002*
Kerala	0.39	*<0*.*001*		0.20	*<0*.*001*	0.19	*<0*.*001*	0.18	*0*.*027*
Tamil Nadu	0.76	*0*.*418*		0.24	*<0*.*001*	0.34	*<0*.*001*	0.41	*0*.*143*
Pondicherry	1.00			0.25	*<0*.*001*	0.56	*0*.*121*		
Andaman and Nicobar	0.03	*<0*.*001*		0.43	*0*.*116*			0.44	*0*.*317*
**Constant**	0.18	*<0*.*001*		0.22	*<0*.*001*	0.30	*<0*.*001*	0.12	*<0*.*001*
**Number of obs**	19,276			30,887		14,958		13,292	
**LR chi2(62)**	1872.21			3000		1410.02		1464.51	
**Prob > chi2**	0.00			0		0.00		0.00	
**Pseudo R2**	0.13			0.11		0.10		0.12	
**Log likelihood**	-6327.49			-12022		-6549.00		-5243.18	

LEG: Lower economic group; MEG: Middle economic group; HEG: Higher economic group; OBC: Other Backward Class; SC: Scheduled Castes; ST: Scheduled Tribes; OR denotes odds ratio; ® denotes the reference category.

### Association between drought and temporary migration across the different socioeconomic groups

There is a positive association between drought and temporary migration in the overall sample (OR 1.64 with p<1%) as well as among the socio-economically marginalised sections compared to their better off counterparts after controlling all the covariates (Tables 3 and 4). Households in the LEG have more than two times (OR 2.32 with p<1%) likelihood for having at least one temporary migrant member when they are based at a drought affected district in comparison to a district not affected by drought while controlling all other variables. This ratio decreases to one and half times (OR 1.55 with p<1%) for households belonging to the MEG and the association renders statistically insignificant when HEG is concerned. Among the households belonging to OBC, the probability for having at least one migrant member is almost two times greater (OR 1.95 with p<1%) when they belong to a drought affected district in comparison to a non-drought affected district. This ratio is 1.92 for SC and 1.59 for ST with less than 1% p-value. The association between drought and migration for the Others category households is statistically insignificant. The association between flood and migration is insignificant for all the categories except SC (OR 0.86 with p<10%) and ST (OR 1.74 with p<1%).

### Robustness checks

For checking the consistency of the results of binary logistic regression models, multiple linear regression models were fitted onto the same datasets at household level. The results show that the direction of relationship still holds true in the simple OLS models but this time with lesser intensity (S9 Table in S1 File). There is a positive association between drought and probability of a household to have at least one temporary migrant member (OR 1.05 with p<1%). This association holds positive for households in the marginal socio-economic categories like LEG (OR 1.17 with p<1%), MEG (OR 1.05 with p<1%), ST (OR 1.05 with p<1%), SC (OR 1.09 with p<1%) and OBC (OR 1.09 with p<1%) and statistically insignificant for HEG and Others.

Again datasets were prepared for the individual level (rather the household level which has been the unit of analysis till now) for age group 15–64 and binary logistic regression models were fitted (S10 Table in S1 File). At individual level analysis almost the similar relationships between drought and migration hold true as seen for the binary logistic regression models at household level. Drought is positively associated with migration for the overall sample (OR 1.69 with p<1%) and also for the marginalised socio-economic classes of LEG (OR 2.36 with p<1%), MEG (OR 1.52 with p<1%), ST (OR 1.76 with p<1%), SC (OR 1.81 with p<1%) and OBC (OR 1.90 with p<1%) and not significant for HEG and Others.

The criteria for drought definition for the study is set till now as at least 40% negative deviation in the annual rainfall from the normal rainfall for the district. Household level binary logistic regression models were also constructed for other measures of drought with 10% to 60% negative annual rainfall deviations at every 5% intervals. Other than 40% negative annual rainfall deviation measure of drought, the only other measure which produces statistically significant relationships between drought and temporary migration is 35% negative annual deviation but this time with lesser intensity.

## Discussion

The study investigates the association between drought occurrence and probability of a household to have at least one temporary migrant member with NSSO 64^th^ round data and IMD data fitted into quantitative models. This is one of the few studies especially on the Indian population where association between temporary migration and a specific natural hazard (here drought) has been investigated as against the dominant trend of using one or the other constructs of climate change or climate variability [30, 52, 53]. This association has been assessed at household level and across the different socioeconomic groups in rural India after combining the objective meteorological data from IMD with NSSO sample survey data. Out of the total temporary migrants in rural India, 99.46% migrated within the national border and 67.12% were rural to urban migrants. The study reveals a strong association between occurrence of drought and the probability of a household having at least one temporary migrant member. This finding is consistent with a study [28] which has found that drought frequency in the origin state brings with it an increase in the inter-state migration in India by analysing Census of India data 1991 and 2001. Again both the studies did not find any statistical association between flood (“excess precipitation”) and migration. A similar finding also has come up from another study [29] which with the exploration of ICRISAT cross-sectional data of 2013–14 found that climatic risks (which was reported to be heavily related to drought) brings an increase in the probability of migration in semi-arid parts of India. Real and futuristic migration intentions data may produce somewhat contradictory associations with drought as one study [54] employing perceived increased severity of drought and future migration intentions, has reported a negative association between the two.

The positive association between drought and migration may be explained with the help of conceptual frameworks proposed in the literature time to time. Migration may be influenced by a range of drivers including economic, social, political, demographic and environmental [47]. Drought may either directly influence migration through squeezing the ecosystem services (like increasing water shortages [55]) or through manipulating the other drivers of migration (like decreasing crop yield [8, 9] or through intensifying social conflicts and decaying local social capitals [56]). These cumulative effects of drivers being filtered through other personal, household/community level factors may produce a migration. Another contribution of the study is the consideration of household as the unit of analysis. As per one of a theoretical strands of New Economics of Labour Migration [57] a household may distribute its workforce across diversified geographies/sectors as a risk aversive insurance strategy as if one geography/sector suffers from any crisis, the remittances from the other geography/sector(s) may keep the household going.

The study reveals a variation in the association between drought and migration across different socio-economic groups where socio-economically marginalised households have more probability for migration than their socio-economically better-off counterparts. This finding is consistent with a few field-based studies which point out that the decisions of migration and non-migration during the instances of droughts are very much influenced by the different socioeconomic backgrounds of the people in India [7, 32, 33, 35, 36, 39, 40, 42]. An explanation through the lens of intersectionality may produce some relevant interpretation in this regard. An intersectional analysis illuminates how different households and groups relate differently to drought, due to their situatedness in power structures based on context-specific and dynamic social categorisations [58]. Drought may be experienced differently by different socioeconomic groups based on their different sets of resource availability and different adaptation/coping strategies. For relatively better socially and/or economically endowed households in-situ adaptation may be more preferred, but for poorly endowed households migration may be a result of failure to adapt in-situ or migration itself may be an opportunity for adaptation to drought if planned properly [59].

An examination of the association between temporary migration and covariates reveals that temporary migration as a livelihood strategy is highly concentrated among the socioeconomically marginalised sections of Indian rural population. It is the households belonging to marginalised social groups, households with smaller land possessions, households with main income source from agricultural labour and other labour, households with lower MPCE levels and households having illiterate heads are the ones with greater probability for having temporary migrant members. Various previous studies also had come up with similar findings [36, 49, 50, 60–64]. Mere the occurrence of these socioeconomic drivers of temporary migration (i.e. belonging to marginalised sections) may not be enough to cause temporary migration to happen. There are certain other household characteristics or prerequisites which may facilitate a household to undertake a decision on temporary migration that this study has identified like households with more than 5 members, households without any out-migrant member, households affiliated with Islam, male headed households, households with head aged between 45–54 years and widowed headed households. It may be argued that having a minimum household size may facilitate a household to retain enough workforce at home while a section of it goes out of the village temporarily. Many of these prerequisites of migration have also come up in other studies from time to time [47, 65].

The states of Uttar Pradesh, Andhra Pradesh, Bihar, West Bengal, Maharashtra etc. are the home to rural population who majorly belong to socioeconomically disadvantaged sections and this makes these states greater contributor of temporary migrants in India. Deprivations in the historical lines as well as lacuna in the present policy interventions may explain the production and reproduction of marginality in rural areas of these states. One of the processes of creating marginalisation may be exclusion from the traditional livelihood asset base like forest evection especially in the case of ST population [66]. In contrast to permanent and semi-permanent migrants who are drawn from more affluent parts of the community, temporary migration is mostly used by this impoverished and deprived socioeconomic segments of the Indian population as a technique to alleviate poverty [67]. However, temporary migration continues to be a costly and risky endeavour in terms of both tagibilities and intangibilities [65, 68]. This may be the reason why initiatives to increase rural employment, like NREGA, may be able to reduce temporary migration even if they only compensate part of the economic benefits gained through temporary migration [69]. However, a few research also suggest that temporary migration is not just a survival strategy of poor people [70, 71]. They contend that temporary migration can act as both a shelter of liberation and a path towards accumulation.

The study is not without limitations. Absence of any recent government dataset on temporary migration made the analysis dependent on a dataset (NSSO 64^th^ round) which was surveyed 13/14 years back and this may be the prime limitation of the study. One of the important limitations are related to the measurement issues of both migration and drought. The definition of temporary migration used in NSSO is restricted only to a very short time duration (away from the village for 1 month or more but less than 6 months during last 365 days) where a relaxation of time may have included a much broader picture. Consideration of more advanced measurements of drought in terms of Standardised Precipitation Index (SPI) or Standardised Precipitation Evapotranspiration Index (SPEI) or consideration of soil moisture drought or agricultural drought would have provided a more grounded analysis. Another dimension of measurement issue is the temporal discrepancy between drought and migration as drought is measured on a yearly basis which ranges between 1^st^ of January to 31^st^ of December and migration between 1^st^ of July to 30^th^ of June. One important limitation about model specification is absence of pull factors. The type of econometric model used in the study makes the results limited to correlational conclusions only rather than causation between drought and temporary migration. Some more advanced econometric models like instrumental variable models or structural equation models may serve this purpose. Here one thing needs mentioning is that in the study almost half of the districts affected with drought are concentrated in a single state of Tamil Nadu itself as the state was hit by a severe drought in 2003. A different geographical pattern of drought may have produced a different drought migration nexus. It is out of scope for this analysis to say whether this temporary migration in relation to drought is forced (displacement) or voluntary (planned migration). Again its also not possible to say whether this migration is an adaptation to drought or a failure to adaptation to drought as for this type of conclusions impact data of migration needs to be analysed.

## Conclusion

The study finds that there is a positive association between drought instances and the probability of a household to have at least one temporary migrant member in rural India by fitting binary logistic regression models on a dataset involving NSSO 64^th^ round data and IMD rainfall data while controlling all other confounding variables. The study also concludes that the probability of temporary migration on account of drought is more among the socio-economically marginalised sections of the society compared to their better-off counterparts.

## Supporting information

S1 File (DOCX)
