# Supplementary material for: Drought and temporary migration in rural India: A comparative study across different socio-economic groups with a cross-sectional nationally representative dataset
Source: PLoS One. 2022 Oct 7;17(10):e0275449. doi: 10.1371/journal.pone.0275449 (PMC9683024; doi:10.1371/journal.pone.0275449)
Supplement: S1 File — (DOCX) [file pone.0275449.s001.docx]

**S1 Table. Pairwise correlation coefficient matrix of explanatory variables for overall sample.**

| **Explanatory variables** | **1** | **2** | **3** | **4** | **5** | **6** | **7** | **8** | **9** | **10** | **11** | **12** | **13** | **14** |
| --- | --- | --- | --- | --- | --- | --- | --- | --- | --- | --- | --- | --- | --- | --- |
| **1.Drought** | 1.00 |  |  |  |  |  |  |  |  |  |  |  |  |  |
|  |  |  |  |  |  |  |  |  |  |  |  |  |  |  |
| **2.Flood** | 0.11 | 1.00 |  |  |  |  |  |  |  |  |  |  |  |  |
|  | *<0.001* |  |  |  |  |  |  |  |  |  |  |  |  |  |
| **3.Household size** | -0.06 | 0.00 | 1.00 |  |  |  |  |  |  |  |  |  |  |  |
|  | *<0.001* | *0.264* |  |  |  |  |  |  |  |  |  |  |  |  |
| **4.Household with out-migrant** | 0.01 | 0.02 | -0.01 | 1.00 |  |  |  |  |  |  |  |  |  |  |
|  | *0.007* | *<0.001* | *<0.001* |  |  |  |  |  |  |  |  |  |  |  |
| **5.Religion** | 0.12 | -0.04 | 0.04 | 0.02 | 1.00 |  |  |  |  |  |  |  |  |  |
|  | *<0.001* | *<0.001* | *<0.001* | *<0.001* |  |  |  |  |  |  |  |  |  |  |
| **6.Social group** | 0.07 | 0.02 | 0.01 | 0.08 | 0.18 | 1.00 |  |  |  |  |  |  |  |  |
|  | *<0.001* | *<0.001* | *0.001* | *<0.001* | *<0.001* |  |  |  |  |  |  |  |  |  |
| **7.Land possession** | -0.03 | -0.01 | 0.16 | -0.10 | 0.03 | -0.04 | 1.00 |  |  |  |  |  |  |  |
|  | *<0.001* | *0.131* | *<0.001* | *<0.001* | *<0.001* | *<0.001* |  |  |  |  |  |  |  |  |
| **8.Occupation type** | 0.06 | 0.00 | -0.16 | 0.02 | -0.02 | 0.01 | -0.36 | 1.00 |  |  |  |  |  |  |
|  | *<0.001* | *0.318* | *<0.001* | *<0.001* | *<0.001* | *0.008* | *<0.001* |  |  |  |  |  |  |  |
| **9.MPCE tertiles** | 0.05 | -0.01 | -0.26 | -0.12 | 0.15 | -0.14 | 0.11 | 0.05 | 1.00 |  |  |  |  |  |
|  | *<0.001* | *<0.001* | *<0.001* | *<0.001* | *<0.001* | *<0.001* | *<0.001* | *<0.001* |  |  |  |  |  |  |
| **10.Sex** | 0.03 | -0.01 | -0.15 | -0.22 | 0.00 | -0.02 | -0.10 | 0.20 | 0.06 | 1.00 |  |  |  |  |
|  | *<0.001* | *0.130* | *<0.001* | *<0.001* | *0.377* | *<0.001* | *<0.001* | *<0.001* | *<0.001* |  |  |  |  |  |
| **11.Age** | 0.02 | -0.01 | 0.17 | -0.30 | -0.01 | -0.08 | 0.14 | -0.09 | 0.08 | 0.00 | 1.00 |  |  |  |
|  | *<0.001* | *0.006* | *<0.001* | *<0.001* | *0.078* | *<0.001* | *<0.001* | *<0.001* | *<0.001* | *0.169* |  |  |  |  |
| **12.Marital status** | 0.04 | 0.00 | -0.10 | -0.06 | -0.01 | 0.00 | -0.05 | 0.09 | 0.06 | 0.55 | 0.21 | 1.00 |  |  |
|  | *<0.001* | *0.610* | *<0.001* | *<0.001* | *<0.001* | *0.322* | *<0.001* | *<0.001* | *<0.001* | *<0.001* | *<0.001* |  |  |  |
| **13.Educational level** | 0.02 | -0.02 | -0.06 | 0.06 | 0.06 | -0.14 | 0.08 | 0.08 | 0.30 | -0.17 | -0.19 | -0.19 | 1.00 |  |
|  | *<0.001* | *<0.001* | *<0.001* | *<0.001* | *<0.001* | *<0.001* | *<0.001* | *<0.001* | *<0.001* | *<0.001* | *<0.001* | *<0.001* |  |  |
| **14.State** | 0.28 | 0.01 | -0.15 | 0.00 | -0.11 | 0.03 | 0.02 | 0.10 | 0.02 | 0.02 | 0.05 | 0.07 | 0.02 | 1.00 |
|  | *<0.001* | *0.139* | *<0.001* | *0.854* | *<0.001* | *<0.001* | *<0.001* | *<0.001* | *<0.001* | *<0.001* | *<0.001* | *<0.001* | *<0.001* |  |

Note: First row has correlation coefficient value and second row has associated level of significance.

**S2 Table. Pairwise correlation coefficient matrix of explanatory variables for lower economic group sample.**

| **Explanatory variables** | **1** | **2** | **3** | **4** | **5** | **6** | **7** | **8** | **9** | **10** | **11** | **12** | **13** |
| --- | --- | --- | --- | --- | --- | --- | --- | --- | --- | --- | --- | --- | --- |
| **1.Drought** | 1.00 |  |  |  |  |  |  |  |  |  |  |  |  |
|  |  |  |  |  |  |  |  |  |  |  |  |  |  |
| **2.Flood** | 0.10 | 1.00 |  |  |  |  |  |  |  |  |  |  |  |
|  | *<0.001* |  |  |  |  |  |  |  |  |  |  |  |  |
| **3.Household size** | -0.06 | 0.00 | 1.00 |  |  |  |  |  |  |  |  |  |  |
|  | *<0.001* | *0.562* |  |  |  |  |  |  |  |  |  |  |  |
| **4.Household with out-migrant** | 0.02 | -0.01 | -0.04 | 1.00 |  |  |  |  |  |  |  |  |  |
|  | *0.002* | *0.157* | *<0.001* |  |  |  |  |  |  |  |  |  |  |
| **5.Religion** | 0.05 | -0.04 | 0.05 | 0.01 | 1.00 |  |  |  |  |  |  |  |  |
|  | *<0.001* | *<0.001* | *<0.001* | *0.127* |  |  |  |  |  |  |  |  |  |
| **6.Social group** | 0.04 | 0.05 | -0.06 | 0.06 | -0.04 | 1.00 |  |  |  |  |  |  |  |
|  | *<0.001* | *<0.001* | *<0.001* | *<0.001* | *<0.001* |  |  |  |  |  |  |  |  |
| **7.Land possession** | -0.02 | -0.04 | 0.17 | -0.08 | -0.03 | 0.00 | 1.00 |  |  |  |  |  |  |
|  | *0.005* | *<0.001* | *<0.001* | *<0.001* | *<0.001* | *0.625* |  |  |  |  |  |  |  |
| **8.Occupation type** | 0.07 | 0.01 | -0.18 | 0.02 | -0.01 | 0.03 | -0.38 | 1.00 |  |  |  |  |  |
|  | *<0.001* | *0.021* | *<0.001* | *<0.001* | *0.041* | *<0.001* | *<0.001* |  |  |  |  |  |  |
| **9.Sex** | 0.02 | 0.01 | -0.16 | -0.21 | 0.01 | -0.03 | -0.08 | 0.19 | 1.00 |  |  |  |  |
|  | *<0.001* | *0.096* | *<0.001* | *<0.001* | *0.124* | *<0.001* | *<0.001* | *<0.001* |  |  |  |  |  |
| **10.Age** | 0.02 | -0.01 | 0.22 | -0.25 | -0.02 | -0.08 | 0.12 | -0.09 | 0.01 | 1.00 |  |  |  |
|  | *0.001* | *0.095* | *<0.001* | *<0.001* | *0.009* | *<0.001* | *<0.001* | *<0.001* | *0.159* |  |  |  |  |
| **11.Marital status** | 0.03 | -0.02 | -0.09 | -0.05 | -0.01 | 0.00 | -0.03 | 0.08 | 0.56 | 0.23 | 1.00 |  |  |
|  | *<0.001* | *0.008* | *<0.001* | *<0.001* | *0.252* | *0.884* | *<0.001* | *<0.001* | *<0.001* | *<0.001* |  |  |  |
| **12.Educational level** | 0.01 | -0.01 | 0.00 | 0.08 | -0.01 | -0.11 | 0.07 | -0.08 | -0.19 | -0.19 | -0.16 | 1.00 |  |
|  | *0.048* | *0.310* | *0.836* | *<0.001* | *0.091* | *<0.001* | *<0.001* | *<0.001* | *<0.001* | *<0.001* | *<0.001* |  |  |
| **13.State** | 0.27 | -0.14 | -0.16 | 0.00 | -0.05 | 0.09 | 0.11 | 0.06 | 0.01 | 0.07 | 0.08 | 0.02 | 1.00 |
|  | *<0.001* | *<0.001* | *<0.001* | *0.687* | *<0.001* | *<0.001* | *<0.001* | *<0.001* | *0.322* | *<0.001* | *<0.001* | *0.001* |  |

Note: First row has correlation coefficient value and second row has associated level of significance.

**S3 Table. Pairwise correlation coefficient matrix of explanatory variables for middle economic group sample.**

| **Explanatory variables** | **1** | **2** | **3** | **4** | **5** | **6** | **7** | **8** | **9** | **10** | **11** | **12** | **13** |
| --- | --- | --- | --- | --- | --- | --- | --- | --- | --- | --- | --- | --- | --- |
| **1.Drought** | 1.00 |  |  |  |  |  |  |  |  |  |  |  |  |
|  |  |  |  |  |  |  |  |  |  |  |  |  |  |
| **2.Flood** | 0.13 | 1.00 |  |  |  |  |  |  |  |  |  |  |  |
|  | *<0.001* |  |  |  |  |  |  |  |  |  |  |  |  |
| **3.Household size** | -0.04 | 0.01 | 1.00 |  |  |  |  |  |  |  |  |  |  |
|  | *<0.001* | *0.113* |  |  |  |  |  |  |  |  |  |  |  |
| **4.Household with out-migrant** | 0.03 | 0.03 | -0.05 | 1.00 |  |  |  |  |  |  |  |  |  |
|  | *<0.001* | *<0.001* | *<0.001* |  |  |  |  |  |  |  |  |  |  |
| **5.Religion** | 0.19 | 0.00 | 0.09 | 0.06 | 1.00 |  |  |  |  |  |  |  |  |
|  | *<0.001* | *0.762* | *<0.001* | *<0.001* |  |  |  |  |  |  |  |  |  |
| **6.Social group** | 0.13 | 0.04 | -0.03 | 0.04 | 0.20 | 1.00 |  |  |  |  |  |  |  |
|  | *<0.001* | *<0.001* | *<0.001* | *<0.001* | *<0.001* |  |  |  |  |  |  |  |  |
| **7.Land possession** | -0.04 | 0.01 | 0.21 | -0.08 | -0.02 | -0.05 | 1.00 |  |  |  |  |  |  |
|  | *<0.001* | *0.289* | *<0.001* | *<0.001* | *<0.001* | *<0.001* |  |  |  |  |  |  |  |
| **8.Occupation type** | 0.06 | 0.00 | -0.16 | 0.01 | -0.03 | 0.03 | -0.36 | 1.00 |  |  |  |  |  |
|  | *<0.001* | *0.779* | *<0.001* | *0.051* | *<0.001* | *<0.001* | *<0.001* |  |  |  |  |  |  |
| **9.Sex** | 0.03 | -0.01 | -0.13 | -0.20 | 0.00 | 0.01 | -0.11 | 0.20 | 1.00 |  |  |  |  |
|  | *<0.001* | *0.101* | *<0.001* | *<0.001* | *0.518* | *0.256* | *<0.001* | *<0.001* |  |  |  |  |  |
| **10.Age** | 0.02 | -0.01 | 0.21 | -0.30 | -0.02 | -0.05 | 0.12 | -0.09 | 0.01 | 1.00 |  |  |  |
|  | *0.006* | *0.355* | *<0.001* | *<0.001* | *<0.001* | *<0.001* | *<0.001* | *<0.001* | *0.204* |  |  |  |  |
| **11.Marital status** | 0.04 | 0.00 | -0.09 | -0.06 | -0.01 | 0.02 | -0.06 | 0.10 | 0.58 | 0.21 | 1.00 |  |  |
|  | *<0.001* | *0.952* | *<0.001* | *<0.001* | *0.033* | *<0.001* | *<0.001* | *<0.001* | *<0.001* | *<0.001* |  |  |  |
| **12.Educational level** | 0.01 | -0.01 | 0.05 | 0.10 | 0.02 | -0.12 | 0.07 | 0.00 | -0.21 | -0.21 | -0.21 | 1.00 |  |
|  | *0.053* | *0.241* | *<0.001* | *<0.001* | *0.007* | *<0.001* | *<0.001* | *0.826* | *<0.001* | *<0.001* | *<0.001* |  |  |
| **13.State** | 0.26 | 0.04 | -0.16 | 0.00 | -0.10 | 0.04 | 0.03 | 0.12 | 0.04 | 0.05 | 0.07 | -0.01 | 1.00 |
|  | *<0.001* | *<0.001* | *<0.001* | *0.860* | *<0.001* | *<0.001* | *<0.001* | *<0.001* | *<0.001* | *<0.001* | *<0.001* | *0.044* |  |

Note: First row has correlation coefficient value and second row has associated level of significance.

**S4 Table. Pairwise correlation coefficient matrix of explanatory variables for higher economic group sample.**

| **Explanatory variables** | **1** | **2** | **3** | **4** | **5** | **6** | **7** | **8** | **9** | **10** | **11** | **12** | **13** |
| --- | --- | --- | --- | --- | --- | --- | --- | --- | --- | --- | --- | --- | --- |
| **1.Drought** | 1.00 |  |  |  |  |  |  |  |  |  |  |  |  |
|  |  |  |  |  |  |  |  |  |  |  |  |  |  |
| **2.Flood** | 0.11 | 1.00 |  |  |  |  |  |  |  |  |  |  |  |
|  | *<0.001* |  |  |  |  |  |  |  |  |  |  |  |  |
| **3.Household size** | -0.04 | -0.01 | 1.00 |  |  |  |  |  |  |  |  |  |  |
|  | *<0.001* | *0.378* |  |  |  |  |  |  |  |  |  |  |  |
| **4.Household with out-migrant** | 0.00 | 0.03 | -0.05 | 1.00 |  |  |  |  |  |  |  |  |  |
|  | *0.856* | *<0.001* | *<0.001* |  |  |  |  |  |  |  |  |  |  |
| **5.Religion** | 0.09 | -0.06 | 0.12 | 0.05 | 1.00 |  |  |  |  |  |  |  |  |
|  | *<0.001* | *<0.001* | *<0.001* | *<0.001* |  |  |  |  |  |  |  |  |  |
| **6.Social group** | 0.07 | -0.01 | 0.03 | 0.08 | 0.35 | 1.00 |  |  |  |  |  |  |  |
|  | *<0.001* | *0.052* | *<0.001* | *<0.001* | *<0.001* |  |  |  |  |  |  |  |  |
| **7.Land possession** | -0.06 | 0.01 | 0.22 | -0.11 | 0.06 | -0.02 | 1.00 |  |  |  |  |  |  |
|  | *<0.001* | *0.020* | *<0.001* | *<0.001* | *<0.001* | *0.005* |  |  |  |  |  |  |  |
| **8.Occupation type** | 0.05 | 0.00 | -0.13 | 0.05 | -0.04 | -0.01 | -0.37 | 1.00 |  |  |  |  |  |
|  | *<0.001* | *0.655* | *<0.001* | *<0.001* | *<0.001* | *0.295* | *<0.001* |  |  |  |  |  |  |
| **9.Sex** | 0.03 | -0.01 | -0.12 | -0.23 | -0.03 | -0.01 | -0.14 | 0.20 | 1.00 |  |  |  |  |
|  | *<0.001* | *0.032* | *<0.001* | *<0.001* | *<0.001* | *0.185* | *<0.001* | *<0.001* |  |  |  |  |  |
| **10.Age** | 0.02 | -0.01 | 0.17 | -0.34 | -0.02 | -0.06 | 0.14 | -0.11 | -0.01 | 1.00 |  |  |  |
|  | *0.002* | *0.094* | *<0.001* | *<0.001* | *0.009* | *<0.001* | *<0.001* | *<0.001* | *0.022* |  |  |  |  |
| **11.Marital status** | 0.04 | 0.01 | -0.09 | -0.05 | -0.04 | 0.01 | -0.08 | 0.08 | 0.51 | 0.19 | 1.00 |  |  |
|  | *<0.001* | *0.064* | *<0.001* | *<0.001* | *<0.001* | *0.115* | *<0.001* | *<0.001* | *<0.001* | *<0.001* |  |  |  |
| **12.Educational level** | 0.00 | -0.02 | -0.01 | 0.14 | 0.02 | -0.10 | 0.01 | 0.21 | -0.20 | -0.26 | -0.25 | 1.00 |  |
|  | *0.830* | *0.001* | *0.083* | *<0.001* | *0.001* | *<0.001* | *0.017* | *<0.001* | *<0.001* | *<0.001* | *<0.001* |  |  |
| **13.State** | 0.31 | 0.12 | -0.14 | 0.01 | -0.16 | -0.03 | -0.06 | 0.11 | 0.02 | 0.02 | 0.05 | 0.03 | 1.00 |
|  | *<0.001* | *<0.001* | *<0.001* | *0.177* | *<0.001* | *<0.001* | *<0.001* | *<0.001* | *0.002* | *0.003* | *<0.001* | *<0.001* |  |

Note: First row has correlation coefficient value and second row has associated level of significance.

**S5 Table. Pairwise correlation coefficient matrix of explanatory variables for Others social group sample.**

| **Explanatory variables** | **1** | **2** | **3** | **4** | **5** | **6** | **7** | **8** | **9** | **10** | **11** | **12** | **13** |
| --- | --- | --- | --- | --- | --- | --- | --- | --- | --- | --- | --- | --- | --- |
| **1.Drought** | 1.00 |  |  |  |  |  |  |  |  |  |  |  |  |
|  |  |  |  |  |  |  |  |  |  |  |  |  |  |
| **2.Flood** | -0.02 | 1.00 |  |  |  |  |  |  |  |  |  |  |  |
|  | *0.028* |  |  |  |  |  |  |  |  |  |  |  |  |
| **3.Household size** | -0.02 | 0.00 | 1.00 |  |  |  |  |  |  |  |  |  |  |
|  | *0.001* | *0.595* |  |  |  |  |  |  |  |  |  |  |  |
| **4.Household with out-migrant** | 0.01 | 0.03 | -0.03 | 1.00 |  |  |  |  |  |  |  |  |  |
|  | *0.205* | *<0.001* | *<0.001* |  |  |  |  |  |  |  |  |  |  |
| **5.Religion** | 0.16 | -0.06 | 0.06 | 0.03 | 1.00 |  |  |  |  |  |  |  |  |
|  | *<0.001* | *<0.001* | *<0.001* | *<0.001* |  |  |  |  |  |  |  |  |  |
| **6.Land possession** | -0.01 | 0.04 | 0.17 | -0.12 | -0.07 | 1.00 |  |  |  |  |  |  |  |
|  | *0.245* | *<0.001* | *<0.001* | *<0.001* | *<0.001* |  |  |  |  |  |  |  |  |
| **7.Occupation type** | 0.02 | -0.02 | -0.18 | 0.05 | 0.03 | -0.37 | 1.00 |  |  |  |  |  |  |
|  | *0.005* | *0.006* | *<0.001* | *<0.001* | *<0.001* | *<0.001* |  |  |  |  |  |  |  |
| **8.MPCE tertiles** | 0.08 | 0.04 | -0.28 | -0.11 | -0.02 | 0.10 | 0.08 | 1.00 |  |  |  |  |  |
|  | *<0.001* | *<0.001* | *<0.001* | *<0.001* | *0.011* | *<0.001* | *<0.001* |  |  |  |  |  |  |
| **9.Sex** | -0.01 | -0.02 | -0.15 | -0.21 | -0.01 | -0.12 | 0.20 | 0.06 | 1.00 |  |  |  |  |
|  | *0.162* | *0.001* | *<0.001* | *<0.001* | *0.340* | *<0.001* | *<0.001* | *<0.001* |  |  |  |  |  |
| **10.Age** | 0.02 | 0.00 | 0.21 | -0.33 | -0.04 | 0.15 | -0.10 | 0.06 | -0.01 | 1.00 |  |  |  |
|  | *0.027* | *0.669* | *<0.001* | *<0.001* | *<0.001* | *<0.001* | *<0.001* | *<0.001* | *0.194* |  |  |  |  |
| **11.Marital status** | 0.00 | -0.01 | -0.08 | -0.06 | -0.02 | -0.06 | 0.10 | 0.04 | 0.54 | 0.20 | 1.00 |  |  |
|  | *0.721* | *0.077* | *<0.001* | *<0.001* | *0.010* | *<0.001* | *<0.001* | *<0.001* | *<0.001* | *<0.001* |  |  |  |
| **12.Educational level** | 0.03 | 0.03 | -0.10 | 0.06 | -0.14 | 0.07 | 0.11 | 0.28 | -0.19 | -0.18 | -0.20 | 1.00 |  |
|  | *<0.001* | *<0.001* | *<0.001* | *<0.001* | *<0.001* | *<0.001* | *<0.001* | *<0.001* | *<0.001* | *<0.001* | *<0.001* |  |  |
| **13.State** | 0.07 | -0.06 | -0.13 | 0.01 | -0.06 | 0.00 | 0.09 | -0.05 | 0.00 | 0.01 | 0.03 | -0.01 | 1.00 |
|  | *<0.001* | *<0.001* | *<0.001* | *0.351* | *<0.001* | *0.579* | *<0.001* | *<0.001* | *0.583* | *0.144* | *<0.001* | *0.144* |  |

Note: First row has correlation coefficient value and second row has associated level of significance.

**S6 Table. Pairwise correlation coefficient matrix of explanatory variables for Other Backward Class sample.**

| **Explanatory variables** | **1** | **2** | **3** | **4** | **5** | **6** | **7** | **8** | **9** | **10** | **11** | **12** | **13** |
| --- | --- | --- | --- | --- | --- | --- | --- | --- | --- | --- | --- | --- | --- |
| **1.Drought** | 1.00 |  |  |  |  |  |  |  |  |  |  |  |  |
|  |  |  |  |  |  |  |  |  |  |  |  |  |  |
| **2.Flood** | 0.13 | 1.00 |  |  |  |  |  |  |  |  |  |  |  |
|  | *<0.001* |  |  |  |  |  |  |  |  |  |  |  |  |
| **3.Household size** | -0.12 | -0.01 | 1.00 |  |  |  |  |  |  |  |  |  |  |
|  | *<0.001* | *0.119* |  |  |  |  |  |  |  |  |  |  |  |
| **4.Household with out-migrant** | -0.01 | 0.02 | -0.02 | 1.00 |  |  |  |  |  |  |  |  |  |
|  | *0.249* | *<0.001* | *0.001* |  |  |  |  |  |  |  |  |  |  |
| **5.Religion** | 0.05 | -0.02 | 0.05 | 0.00 | 1.00 |  |  |  |  |  |  |  |  |
|  | *<0.001* | *0.002* | *<0.001* | *0.570* |  |  |  |  |  |  |  |  |  |
| **6.Land possession** | -0.07 | -0.01 | 0.16 | -0.10 | -0.09 | 1.00 |  |  |  |  |  |  |  |
|  | *<0.001* | *0.040* | *<0.001* | *<0.001* | *<0.001* |  |  |  |  |  |  |  |  |
| **7.Occupation type** | 0.11 | 0.02 | -0.18 | 0.01 | 0.09 | -0.37 | 1.00 |  |  |  |  |  |  |
|  | *<0.001* | *<0.001* | *<0.001* | *0.035* | *<0.001* | *<0.001* |  |  |  |  |  |  |  |
| **8.MPCE tertiles** | 0.08 | 0.00 | -0.29 | -0.12 | 0.04 | 0.09 | 0.06 | 1.00 |  |  |  |  |  |
|  | *<0.001* | *0.517* | *<0.001* | *<0.001* | *<0.001* | *<0.001* | *<0.001* |  |  |  |  |  |  |
| **9.Sex** | 0.06 | 0.00 | -0.16 | -0.23 | 0.05 | -0.11 | 0.22 | 0.06 | 1.00 |  |  |  |  |
|  | *<0.001* | *0.679* | *<0.001* | *<0.001* | *<0.001* | *<0.001* | *<0.001* | *<0.001* |  |  |  |  |  |
| **10.Age** | 0.04 | 0.00 | 0.16 | -0.30 | -0.01 | 0.13 | -0.09 | 0.05 | 0.00 | 1.00 |  |  |  |
|  | *<0.001* | *0.415* | *<0.001* | *<0.001* | *0.020* | *<0.001* | *<0.001* | *<0.001* | *0.561* |  |  |  |  |
| **11.Marital status** | 0.05 | 0.00 | -0.11 | -0.07 | 0.00 | -0.05 | 0.11 | 0.07 | 0.54 | 0.22 | 1.00 |  |  |
|  | *<0.001* | *0.448* | *<0.001* | *<0.001* | *0.765* | *<0.001* | *<0.001* | *<0.001* | *<0.001* | *<0.001* |  |  |  |
| **12.Educational level** | 0.05 | -0.02 | -0.07 | 0.08 | -0.03 | 0.06 | 0.07 | 0.25 | -0.19 | -0.20 | -0.19 | 1.00 |  |
|  | *<0.001* | *<0.001* | *<0.001* | *<0.001* | *<0.001* | *<0.001* | *<0.001* | *<0.001* | *<0.001* | *<0.001* | *<0.001* |  |  |
| **13.State** | 0.43 | 0.01 | -0.21 | -0.03 | -0.08 | 0.02 | 0.15 | 0.15 | 0.05 | 0.07 | 0.09 | 0.08 | 1.00 |
|  | *<0.001* | *0.119* | *<0.001* | *<0.001* | *<0.001* | *<0.001* | *<0.001* | *<0.001* | *<0.001* | *<0.001* | *<0.001* | *<0.001* |  |

Note: First row has correlation coefficient value and second row has associated level of significance.

**S7 Table. Pairwise correlation coefficient matrix of explanatory variables for Scheduled Castes sample.**

| **Explanatory variables** | **1** | **2** | **3** | **4** | **5** | **6** | **7** | **8** | **9** | **10** | **11** | **12** | **13** |
| --- | --- | --- | --- | --- | --- | --- | --- | --- | --- | --- | --- | --- | --- |
| **1.Drought** | 1.00 |  |  |  |  |  |  |  |  |  |  |  |  |
|  |  |  |  |  |  |  |  |  |  |  |  |  |  |
| **2.Flood** | 0.24 | 1.00 |  |  |  |  |  |  |  |  |  |  |  |
|  | *<0.001* |  |  |  |  |  |  |  |  |  |  |  |  |
| **3.Household size** | -0.06 | -0.01 | 1.00 |  |  |  |  |  |  |  |  |  |  |
|  | *<0.001* | *0.101* |  |  |  |  |  |  |  |  |  |  |  |
| **4.Household with out-migrant** | 0.00 | 0.01 | 0.01 | 1.00 |  |  |  |  |  |  |  |  |  |
|  | *0.623* | *0.163* | *0.466* |  |  |  |  |  |  |  |  |  |  |
| **5.Religion** | 0.00 | -0.06 | -0.03 | -0.01 | 1.00 |  |  |  |  |  |  |  |  |
|  | *0.735* | *<0.001* | *0.001* | *0.314* |  |  |  |  |  |  |  |  |  |
| **6.Land possession** | -0.02 | 0.00 | 0.11 | -0.07 | 0.01 | 1.00 |  |  |  |  |  |  |  |
|  | *0.029* | *0.801* | *<0.001* | *<0.001* | *0.259* |  |  |  |  |  |  |  |  |
| **7.Occupation type** | 0.05 | 0.03 | -0.12 | -0.01 | 0.05 | -0.29 | 1.00 |  |  |  |  |  |  |
|  | *<0.001* | *0.001* | *<0.001* | *0.414* | *<0.001* | *<0.001* |  |  |  |  |  |  |  |
| **8.MPCE tertiles** | 0.02 | -0.02 | -0.27 | -0.13 | 0.09 | 0.06 | 0.06 | 1.00 |  |  |  |  |  |
|  | *0.004* | *0.020* | *<0.001* | *<0.001* | *<0.001* | *<0.001* | *<0.001* |  |  |  |  |  |  |
| **9.Sex** | 0.01 | 0.02 | -0.14 | -0.25 | 0.00 | -0.07 | 0.18 | 0.07 | 1.00 |  |  |  |  |
|  | *0.068* | *0.019* | *<0.001* | *<0.001* | *0.656* | *<0.001* | *<0.001* | *<0.001* |  |  |  |  |  |
| **10.Age** | 0.04 | -0.01 | 0.13 | -0.30 | 0.03 | 0.10 | -0.06 | 0.10 | 0.01 | 1.00 |  |  |  |
|  | *<0.001* | *0.250* | *<0.001* | *<0.001* | *0.002* | *<0.001* | *<0.001* | *<0.001* | *0.076* |  |  |  |  |
| **11.Marital status** | 0.03 | 0.01 | -0.11 | -0.07 | -0.01 | -0.03 | 0.07 | 0.07 | 0.54 | 0.23 | 1.00 |  |  |
|  | *<0.001* | *0.359* | *<0.001* | *<0.001* | *0.147* | *<0.001* | *<0.001* | *<0.001* | *<0.001* | *<0.001* |  |  |  |
| **12.Educational level** | 0.02 | -0.01 | -0.03 | 0.11 | 0.05 | 0.03 | 0.07 | 0.21 | -0.17 | -0.21 | -0.17 | 1.00 |  |
|  | *0.027* | *0.524* | *0.001* | *<0.001* | *<0.001* | *0.001* | *<0.001* | *<0.001* | *<0.001* | *<0.001* | *<0.001* |  |  |
| **13.State** | 0.36 | 0.06 | -0.15 | 0.01 | -0.05 | 0.05 | 0.04 | 0.01 | 0.01 | 0.08 | 0.08 | 0.04 | 1.00 |
|  | *<0.001* | *<0.001* | *<0.001* | *0.151* | *<0.001* | *<0.001* | *<0.001* | *0.077* | *0.274* | *<0.001* | *<0.001* | *<0.001* |  |

Note: First row has correlation coefficient value and second row has associated level of significance.

**S8 Table. Pairwise correlation coefficient matrix of explanatory variables for Scheduled Tribes sample.**

| **Explanatory variables** | **1** | **2** | **3** | **4** | **5** | **6** | **7** | **8** | **9** | **10** | **11** | **12** | **13** |
| --- | --- | --- | --- | --- | --- | --- | --- | --- | --- | --- | --- | --- | --- |
| **1.Drought** | 1.00 |  |  |  |  |  |  |  |  |  |  |  |  |
|  |  |  |  |  |  |  |  |  |  |  |  |  |  |
| **2.Flood** | 0.02 | 1.00 |  |  |  |  |  |  |  |  |  |  |  |
|  | *0.008* |  |  |  |  |  |  |  |  |  |  |  |  |
| **3.Household size** | 0.04 | 0.04 | 1.00 |  |  |  |  |  |  |  |  |  |  |
|  | *<0.001* | *<0.001* |  |  |  |  |  |  |  |  |  |  |  |
| **4.Household with out-migrant** | 0.02 | -0.02 | -0.01 | 1.00 |  |  |  |  |  |  |  |  |  |
|  | *0.013* | *0.025* | *0.499* |  |  |  |  |  |  |  |  |  |  |
| **5.Religion** | 0.29 | -0.05 | 0.06 | 0.00 | 1.00 |  |  |  |  |  |  |  |  |
|  | *<0.001* | *<0.001* | *<0.001* | *0.794* |  |  |  |  |  |  |  |  |  |
| **6.Land possession** | 0.03 | -0.06 | 0.16 | -0.09 | 0.12 | 1.00 |  |  |  |  |  |  |  |
|  | *0.001* | *<0.001* | *<0.001* | *<0.001* | *<0.001* |  |  |  |  |  |  |  |  |
| **7.Occupation type** | 0.01 | 0.00 | -0.11 | 0.03 | -0.11 | -0.30 | 1.00 |  |  |  |  |  |  |
|  | *0.464* | *0.953* | *<0.001* | *0.002* | *<0.001* | *<0.001* |  |  |  |  |  |  |  |
| **8.MPCE tertiles** | 0.08 | -0.07 | -0.16 | -0.09 | 0.43 | 0.12 | 0.04 | 1.00 |  |  |  |  |  |
|  | *<0.001* | *<0.001* | *<0.001* | *<0.001* | *<0.001* | *<0.001* | *<0.001* |  |  |  |  |  |  |
| **9.Sex** | 0.05 | -0.01 | -0.12 | -0.17 | -0.02 | -0.10 | 0.16 | 0.08 | 1.00 |  |  |  |  |
|  | *<0.001* | *0.178* | *<0.001* | *<0.001* | *0.034* | *<0.001* | *<0.001* | *<0.001* |  |  |  |  |  |
| **10.Age** | 0.00 | -0.02 | 0.20 | -0.26 | 0.05 | 0.14 | -0.11 | 0.09 | 0.03 | 1.00 |  |  |  |
|  | *0.646* | *0.009* | *<0.001* | *<0.001* | *<0.001* | *<0.001* | *<0.001* | *<0.001* | *<0.001* |  |  |  |  |
| **11.Marital status** | 0.04 | -0.01 | -0.10 | -0.05 | -0.03 | -0.06 | 0.06 | 0.04 | 0.58 | 0.21 | 1.00 |  |  |
|  | *<0.001* | *0.151* | *<0.001* | *<0.001* | *0.002* | *<0.001* | *<0.001* | *<0.001* | *<0.001* | *<0.001* |  |  |  |
| **12.Educational level** | 0.04 | -0.04 | -0.05 | 0.06 | 0.31 | 0.05 | 0.18 | 0.35 | -0.11 | -0.24 | -0.18 | 1.00 |  |
|  | *<0.001* | *<0.001* | *<0.001* | *<0.001* | *<0.001* | *<0.001* | *<0.001* | *<0.001* | *<0.001* | *<0.001* | *<0.001* |  |  |
| **13.State** | -0.10 | -0.01 | -0.05 | 0.03 | -0.48 | -0.05 | 0.09 | -0.30 | 0.01 | -0.05 | 0.02 | -0.17 | 1.00 |
|  | *<0.001* | *0.416* | *<0.001* | *<0.001* | *<0.001* | *<0.001* | *<0.001* | *<0.001* | *0.416* | *<0.001* | *0.028* | *<0.001* |  |

Note: First row has correlation coefficient value and second row has associated level of significance.

**S9 Table. Exp(Coef.) and associated significance levels from the multiple linear regression models assessing the association between explanatory variables and temporary migration across several economic and social groups in rural India, NSSO 64^th^ round (2007-2008).**

| **Explanatory variables** | | **Overall sample** | | **Economic group** | | | | | | **Social group** | | | | | | | |
| --- | --- | --- | --- | --- | --- | --- | --- | --- | --- | --- | --- | --- | --- | --- | --- | --- | --- |
|  |  |  |  | **Lower economic group** | | **Middle economic group** | | **Higher economic group** | | **Others** | | **Other Backward Castes** | | **Scheduled Castes** | | **Scheduled Tribes** | |
|  |  | **exp(Coef.)** | ***P>t*** | **exp(Coef.)** | ***P>t*** | **exp(Coef.)** | ***P>t*** | **exp(Coef.)** | ***P>t*** | **exp(Coef.)** | ***P>t*** | **exp(Coef.)** | ***P>t*** | **exp(Coef.)** | ***P>t*** | **exp(Coef.)** | ***P>t*** |
| **Rainfall variable** | | | | | | | | | | | | | | | | | |
| **Drought** | **No ®** |  |  |  |  |  |  |  |  |  |  |  |  |  |  |  |  |
|  | **Yes** | 1.05 | *<0.001* | 1.17 | *<0.001* | 1.05 | *<0.001* | 1.01 | *0.282* | 1.00 | *0.793* | 1.09 | *<0.001* | 1.09 | *<0.001* | 1.05 | *<0.001* |
| **Flood** | **No ®** |  |  |  |  |  |  |  |  |  |  |  |  |  |  |  |  |
|  | **Yes** | 1.00 | *0.706* | 1.01 | *0.472* | 0.99 | *0.454* | 1.01 | *0.181* | 0.99 | *0.262* | 0.99 | *0.251* | 0.98 | *0.070* | 1.07 | *<0.001* |
| **Household characteristics** | | | | | | | | | | | | | | | | | |
| **Household size** | **Up to 5 ®** |  |  |  |  |  |  |  |  |  |  |  |  |  |  |  |  |
|  | **More than 5** | 1.05 | *<0.001* | 1.05 | *<0.001* | 1.06 | *<0.001* | 1.05 | *<0.001* | 1.05 | *<0.001* | 1.05 | *<0.001* | 1.06 | *<0.001* | 1.06 | *<0.001* |
| **Household with out-migrant** | **Yes ®** |  |  |  |  |  |  |  |  |  |  |  |  |  |  |  |  |
|  | **No** | 1.07 | *<0.001* | 1.08 | *<0.001* | 1.08 | *<0.001* | 1.06 | *<0.001* | 1.07 | *<0.001* | 1.09 | *<0.001* | 1.08 | *<0.001* | 1.05 | *<0.001* |
| **Religion** | **Hinduism ®** |  |  |  |  |  |  |  |  |  |  |  |  |  |  |  |  |
|  | **Islam** | 1.02 | *<0.001* | 1.02 | *0.046* | 1.02 | *0.031* | 1.03 | *<0.001* | 1.04 | *<0.001* | 1.01 | *0.115* | 1.00 | *0.970* | 1.11 | *0.033* |
|  | **Others** | 1.00 | *0.890* | 1.01 | *0.414* | 1.00 | *0.657* | 1.00 | *0.837* | 0.98 | *0.148* | 1.01 | *0.335* | 1.00 | *0.862* | 1.02 | *0.162* |
| **Social group** | **Others ®** |  |  |  |  |  |  |  |  |  |  |  |  |  |  |  |  |
|  | **Other backward castes** | 1.01 | *0.023* | 1.02 | *0.012* | 1.00 | *0.721* | 1.00 | *0.373* |  |  |  |  |  |  |  |  |
|  | **Scheduled castes** | 1.01 | *0.003* | 1.03 | *0.003* | 1.00 | *0.673* | 1.01 | *0.033* |  |  |  |  |  |  |  |  |
|  | **Scheduled tribes** | 1.02 | *0.001* | 1.03 | *0.001* | 1.00 | *0.889* | 1.01 | *0.058* |  |  |  |  |  |  |  |  |
| **Land possession** | **Up to 1 hectare ®** |  |  |  |  |  |  |  |  |  |  |  |  |  |  |  |  |
|  | **1.01 to 4 hectares** | 1.00 | *0.510* | 1.02 | *0.057* | 0.99 | *0.222* | 0.99 | *0.304* | 1.00 | *0.977* | 1.00 | *0.526* | 1.00 | *0.960* | 1.01 | *0.135* |
|  | **More than 4 hectares** | 0.98 | *0.091* | 1.00 | *0.970* | 0.96 | *0.025* | 0.98 | *0.041* | 0.99 | *0.633* | 0.97 | *0.074* | 0.99 | *0.867* | 1.02 | *0.379* |
| **Occupation type** | **Self-employed in agriculture ®** |  |  |  |  |  |  |  |  |  |  |  |  |  |  |  |  |
|  | **Self-employed in non-agriculture** | 1.02 | *<0.001* | 1.03 | *0.004* | 1.03 | *<0.001* | 1.00 | *0.535* | 0.99 | *0.185* | 1.03 | *<0.001* | 1.02 | *0.106* | 1.04 | *0.003* |
|  | **Agricultural labour** | 1.05 | *<0.001* | 1.06 | *<0.001* | 1.06 | *<0.001* | 1.04 | *<0.001* | 1.03 | *<0.001* | 1.06 | *<0.001* | 1.03 | *0.003* | 1.08 | *<0.001* |
|  | **Other labour** | 1.10 | *<0.001* | 1.14 | *<0.001* | 1.12 | *<0.001* | 1.04 | *<0.001* | 1.08 | *<0.001* | 1.10 | *<0.001* | 1.10 | *<0.001* | 1.13 | *<0.001* |
|  | **Others** | 1.00 | *0.578* | 0.98 | *0.055* | 1.00 | *0.533* | 0.99 | *0.038* | 0.99 | *0.304* | 0.99 | *0.219* | 1.00 | *0.701* | 1.01 | *0.275* |
| **MPCE tertiles** | **Lower ®** |  |  |  |  |  |  |  |  |  |  |  |  |  |  |  |  |
|  | **Middle** | 0.96 | *<0.001* |  |  |  |  |  |  | 0.96 | *<0.001* | 0.96 | *<0.001* | 0.95 | *<0.001* | 0.96 | *<0.001* |
|  | **Upper** | 0.95 | *<0.001* |  |  |  |  |  |  | 0.94 | *<0.001* | 0.95 | *<0.001* | 0.94 | *<0.001* | 0.97 | *0.001* |
| **Household head characteristics** | | | | | | | | | | | | | | | | | |
| **Sex** | **Male ®** |  |  |  |  |  |  |  |  |  |  |  |  |  |  |  |  |
|  | **Female** | 0.94 | *<0.001* | 0.90 | *<0.001* | 0.94 | *<0.001* | 0.97 | *<0.001* | 0.96 | *<0.001* | 0.94 | *<0.001* | 0.91 | *<0.001* | 0.97 | *0.010* |
| **Age** | **15-24 ®** |  |  |  |  |  |  |  |  |  |  |  |  |  |  |  |  |
|  | **25-34** | 0.99 | *0.186* | 0.98 | *0.385* | 0.98 | *0.173* | 0.99 | *0.657* | 1.01 | *0.470* | 1.00 | *0.818* | 0.96 | *0.041* | 0.98 | *0.374* |
|  | **35-44** | 0.98 | *0.009* | 0.97 | *0.107* | 0.97 | *0.025* | 0.98 | *0.169* | 1.00 | *0.837* | 0.99 | *0.506* | 0.95 | *0.014* | 0.96 | *0.039* |
|  | **45-54** | 1.02 | *0.027* | 1.01 | *0.449* | 1.01 | *0.470* | 1.03 | *0.018* | 1.05 | *0.008* | 1.03 | *0.078* | 0.99 | *0.685* | 1.02 | *0.362* |
|  | **55-64** | 1.00 | *0.834* | 1.00 | *0.910* | 0.99 | *0.544* | 1.01 | *0.439* | 1.03 | *0.089* | 1.01 | *0.642* | 0.96 | *0.085* | 1.01 | *0.561* |
|  | **Up to 14** | 0.91 | *0.002* | 0.89 | *0.040* | 0.92 | *0.163* | 0.93 | *0.091* | 0.92 | *0.199* | 0.93 | *0.240* | 0.87 | *0.089* | 0.88 | *0.012* |
|  | **65 and above** | 0.96 | *<0.001* | 0.94 | *0.002* | 0.96 | *0.006* | 0.99 | *0.336* | 1.00 | *0.941* | 0.98 | *0.105* | 0.91 | *<0.001* | 0.96 | *0.104* |
| **Marital status** | **Currently married ®** |  |  |  |  |  |  |  |  |  |  |  |  |  |  |  |  |
|  | **Never married** | 0.98 | *0.025* | 1.00 | *0.945* | 0.99 | *0.552* | 0.98 | *0.042* | 0.99 | *0.422* | 0.98 | *0.136* | 0.99 | *0.743* | 0.98 | *0.359* |
|  | **Widowed** | 1.02 | *<0.001* | 1.05 | *<0.001* | 1.02 | *0.083* | 1.01 | *0.278* | 1.01 | *0.420* | 1.03 | *0.001* | 1.04 | *<0.001* | 0.99 | *0.358* |
|  | **Divorced/separated** | 0.98 | *0.270* | 0.98 | *0.564* | 1.01 | *0.623* | 0.96 | *0.078* | 0.98 | *0.506* | 0.98 | *0.490* | 0.98 | *0.629* | 0.97 | *0.382* |
| **Educational level** | **Not literate ®** |  |  |  |  |  |  |  |  |  |  |  |  |  |  |  |  |
|  | **Literate but up to primary** | 0.99 | *0.105* | 1.00 | *0.916* | 0.99 | *0.055* | 1.00 | *0.880* | 1.00 | *0.538* | 0.99 | *0.180* | 1.00 | *0.973* | 0.99 | *0.247* |
|  | **Above primary but up to secondary** | 0.98 | *<0.001* | 0.97 | *<0.001* | 0.98 | *0.001* | 0.99 | *0.133* | 0.99 | *0.103* | 0.98 | *0.001* | 0.97 | *0.006* | 0.98 | *0.010* |
|  | **Above secondary** | 0.97 | *<0.001* | 0.92 | *<0.001* | 0.99 | *0.165* | 0.99 | *0.020* | 0.99 | *0.501* | 0.97 | *<0.001* | 0.97 | *0.035* | 0.95 | *<0.001* |
| **Geographical variable** | | | | | | | | | | | | | | | | | |
| **State** | **Bihar ®** |  |  |  |  |  |  |  |  |  |  |  |  |  |  |  |  |
|  | **Jammu and Kashmir** | 0.95 | *<0.001* | 0.89 | *0.008* | 0.94 | *0.001* | 0.99 | *0.409* | 0.97 | *0.027* | 0.92 | *0.001* | 0.95 | *0.142* | 0.93 | *0.534* |
|  | **Himachal Pradesh** | 0.87 | *<0.001* | 0.86 | *<0.001* | 0.86 | *<0.001* | 0.90 | *<0.001* | 0.87 | *<0.001* | 0.89 | *<0.001* | 0.89 | *<0.001* | 0.91 | *0.030* |
|  | **Punjab** | 0.84 | *<0.001* | 0.74 | *<0.001* | 0.78 | *<0.001* | 0.88 | *<0.001* | 0.88 | *<0.001* | 0.83 | *<0.001* | 0.81 | *<0.001* | 0.79 | *0.262* |
|  | **Chandigarh** | 0.78 | *<0.001* | 0.75 | *0.218* | 0.72 | *0.033* | 0.83 | *<0.001* | 0.80 | *<0.001* | 0.76 | *<0.001* | 0.78 | *0.078* | 0.81 | *0.410* |
|  | **Uttaranchal** | 0.86 | *<0.001* | 0.82 | *<0.001* | 0.84 | *<0.001* | 0.91 | *<0.001* | 0.88 | *<0.001* | 0.83 | *<0.001* | 0.85 | *<0.001* | 0.89 | *0.056* |
|  | **Haryana** | 0.86 | *<0.001* | 0.79 | *<0.001* | 0.84 | *<0.001* | 0.91 | *<0.001* | 0.88 | *<0.001* | 0.84 | *<0.001* | 0.86 | *<0.001* | 0.87 | *0.570* |
|  | **Delhi** | 0.80 | *<0.001* | 0.70 | *0.123* | 0.77 | *<0.001* | 0.84 | *<0.001* | 0.81 | *<0.001* | 0.87 | *0.056* | 0.78 | *<0.001* | 0.88 | *0.728* |
|  | **Rajasthan** | 0.90 | *<0.001* | 0.87 | *<0.001* | 0.91 | *<0.001* | 0.93 | *<0.001* | 0.91 | *<0.001* | 0.89 | *<0.001* | 0.93 | *<0.001* | 0.96 | *0.271* |
|  | **Uttar Pradesh** | 0.96 | *<0.001* | 0.97 | *0.002* | 0.96 | *<0.001* | 0.95 | *<0.001* | 0.95 | *<0.001* | 0.96 | *<0.001* | 0.98 | *0.083* | 1.04 | *0.486* |
|  | **Sikkim** | 0.83 | *<0.001* | 0.74 | *<0.001* | 0.81 | *<0.001* | 0.88 | *<0.001* | 0.81 | *<0.001* | 0.82 | *<0.001* | 0.79 | *<0.001* | 0.91 | *0.014* |
|  | **Arunachal Pradesh** | 0.91 | *<0.001* | 0.81 | *<0.001* | 0.91 | *<0.001* | 1.00 | *0.846* | 1.01 | *0.789* | 0.89 | *0.221* | 0.73 | *<0.001* | 0.98 | *0.682* |
|  | **Nagaland** | 1.13 | *<0.001* | 1.00 | *1.000* | 1.04 | *0.305* | 1.17 | *<0.001* | 1.07 | *0.453* | 0.86 | *0.093* | 0.78 | *0.175* | 1.22 | *<0.001* |
|  | **Manipur** | 0.83 | *<0.001* | 0.79 | *<0.001* | 0.81 | *<0.001* | 0.87 | *<0.001* | 0.87 | *<0.001* | 0.83 | *<0.001* | 0.86 | *0.018* | 0.86 | *<0.001* |
|  | **Mizoram** | 0.84 | *<0.001* | 0.70 | *<0.001* | 0.85 | *<0.001* | 0.87 | *<0.001* | 0.84 | *0.567* | 0.85 | *0.290* | 1.22 | *0.445* | 0.89 | *0.006* |
|  | **Tripura** | 0.89 | *<0.001* | 0.82 | *<0.001* | 0.89 | *<0.001* | 0.95 | *0.001* | 0.92 | *<0.001* | 0.93 | *<0.001* | 0.90 | *<0.001* | 0.94 | *0.084* |
|  | **Meghalaya** | 0.87 | *<0.001* | 0.67 | *<0.001* | 0.88 | *<0.001* | 0.94 | *<0.001* | 1.00 | *0.892* | 0.84 | *0.147* | 0.65 | *0.108* | 0.92 | *0.048* |
|  | **Assam** | 0.92 | *<0.001* | 0.88 | *<0.001* | 0.93 | *<0.001* | 0.96 | *0.003* | 0.91 | *<0.001* | 0.89 | *<0.001* | 0.91 | *0.007* | 1.06 | *0.124* |
|  | **West Bengal** | 0.97 | *<0.001* | 1.01 | *0.497* | 0.94 | *<0.001* | 0.95 | *<0.001* | 0.98 | *0.153* | 0.96 | *0.031* | 0.97 | *0.050* | 1.03 | *0.396* |
|  | **Jharkhand** | 1.00 | *0.581* | 1.00 | *0.950* | 0.99 | *0.572* | 0.95 | *0.007* | 0.98 | *0.470* | 0.98 | *0.055* | 1.08 | *0.001* | 1.08 | *0.056* |
|  | **Orissa** | 0.88 | *<0.001* | 0.88 | *<0.001* | 0.86 | *<0.001* | 0.88 | *<0.001* | 0.86 | *<0.001* | 0.85 | *<0.001* | 0.89 | *<0.001* | 1.00 | *0.922* |
|  | **Chhattisgarh** | 0.88 | *<0.001* | 0.87 | *<0.001* | 0.88 | *<0.001* | 0.91 | *<0.001* | 0.95 | *0.189* | 0.89 | *<0.001* | 1.00 | *0.910* | 0.92 | *0.018* |
|  | **Madhya Pradesh** | 0.91 | *<0.001* | 0.88 | *<0.001* | 0.92 | *<0.001* | 0.93 | *<0.001* | 0.85 | *<0.001* | 0.89 | *<0.001* | 0.96 | *0.023* | 1.01 | *0.871* |
|  | **Gujarat** | 0.92 | *<0.001* | 0.91 | *<0.001* | 0.94 | *<0.001* | 0.94 | *<0.001* | 0.89 | *<0.001* | 0.90 | *<0.001* | 0.91 | *<0.001* | 1.09 | *0.026* |
|  | **Daman and Diu** | 0.84 | *<0.001* | 0.68 | *0.059* | 0.81 | *0.019* | 0.88 | *<0.001* | 0.82 | *<0.001* | 0.86 | *<0.001* | 0.73 | *0.056* | 0.91 | *0.193* |
|  | **Dadra and Nagar Haveli** | 0.76 | *<0.001* | 0.71 | *<0.001* | 0.76 | *<0.001* | 0.83 | *<0.001* | 0.80 | *<0.001* | 0.77 | *<0.001* | 0.77 | *0.325* | 0.84 | *<0.001* |
|  | **Maharashtra** | 0.87 | *<0.001* | 0.87 | *<0.001* | 0.85 | *<0.001* | 0.89 | *<0.001* | 0.85 | *<0.001* | 0.87 | *<0.001* | 0.89 | *<0.001* | 0.94 | *0.112* |
|  | **Andhra Pradesh** | 0.88 | *<0.001* | 0.91 | *<0.001* | 0.87 | *<0.001* | 0.89 | *<0.001* | 0.87 | *<0.001* | 0.87 | *<0.001* | 0.91 | *<0.001* | 1.01 | *0.756* |
|  | **Karnataka** | 0.92 | *<0.001* | 0.95 | *0.001* | 0.90 | *<0.001* | 0.94 | *<0.001* | 0.93 | *<0.001* | 0.92 | *<0.001* | 0.95 | *0.012* | 0.96 | *0.373* |
|  | **Goa** | 0.85 | *<0.001* | 0.79 | *0.022* | 0.83 | *<0.001* | 0.90 | *0.002* | 0.86 | *<0.001* | 0.84 | *0.007* | 0.84 | *0.147* | 0.85 | *0.522* |
|  | **Lakshadweep** | 0.80 | *<0.001* | 0.69 | *0.026* | 0.80 | *0.006* | 0.84 | *<0.001* | 0.82 | *0.215* | 0.74 | *0.382* |  |  | 0.80 | *0.002* |
|  | **Kerala** | 0.84 | *<0.001* | 0.77 | *<0.001* | 0.83 | *<0.001* | 0.90 | *<0.001* | 0.89 | *<0.001* | 0.82 | *<0.001* | 0.81 | *<0.001* | 0.86 | *0.014* |
|  | **Tamil Nadu** | 0.85 | *<0.001* | 0.72 | *<0.001* | 0.88 | *<0.001* | 0.95 | *0.001* | 0.95 | *0.189* | 0.82 | *<0.001* | 0.85 | *<0.001* | 0.90 | *0.147* |
|  | **Pondicherry** | 0.87 | *<0.001* | 0.63 | *<0.001* | 0.83 | *0.001* | 1.00 | *0.914* | 0.80 | *0.484* | 0.82 | *<0.001* | 0.91 | *0.076* |  |  |
|  | **Andaman and Nicobar** | 0.82 | *<0.001* | 0.83 | *0.109* | 0.77 | *<0.001* | 0.87 | *<0.001* | 0.81 | *<0.001* | 0.88 | *0.011* |  |  | 0.91 | *0.261* |
| **Constant** |  | 1.23 | *<0.001* | 1.23 | *<0.001* | 1.20 | *<0.001* | 1.13 | *<0.001* | 1.20 | *<0.001* | 1.24 | *<0.001* | 1.28 | *<0.001* | 1.14 | *0.002* |
| **Number of obs** |  | 78,994 |  | 26,316 |  | 26,342 |  | 26,336 |  | 19,512 |  | 30,974 |  | 15,085 |  | 13,423 |  |
| **F(64, 78929)** |  | 117.72 |  | 32.75 |  | 32.96 |  | 37.09 |  | 33.73 |  | 49.36 |  | 23.80 |  | 25.09 |  |
| **Prob > F** |  | 0.00 |  | 0.00 |  | 0.00 |  | 0.00 |  | 0.00 |  | 0.00 |  | 0.00 |  | 0.00 |  |
| **R-squared** |  | 0.09 |  | 0.07 |  | 0.07 |  | 0.08 |  | 0.10 |  | 0.09 |  | 0.09 |  | 0.10 |  |
| **Adj R-squared** |  | 0.09 |  | 0.07 |  | 0.07 |  | 0.08 |  | 0.09 |  | 0.09 |  | 0.08 |  | 0.10 |  |
| **Root MSE** |  | 0.35 |  | 0.41 |  | 0.34 |  | 0.28 |  | 0.31 |  | 0.35 |  | 0.37 |  | 0.35 |  |

® denotes the reference category.

**S10 Table. Odds ratio and associated significance levels from the binary logistic regression models assessing the association between explanatory variables and temporary migration across several economic and social groups in rural India for age group 15-64 at individual level, NSSO 64th round (2007-2008).**

| **Explanatory variables** | | **Overall sample** | | **Economic group** | | | | | | **Social group** | | | | | | | |
| --- | --- | --- | --- | --- | --- | --- | --- | --- | --- | --- | --- | --- | --- | --- | --- | --- | --- |
|  |  |  |  | **Lower economic group** | | **Middle economic group** | | **Higher economic group** | | **Others** | | **Other Backward Castes** | | **Scheduled Castes** | | **Scheduled Tribes** | |
|  |  | **OR^a^** | ***P>z*** | **OR** | ***P>z*** | **OR** | ***P>z*** | **OR** | ***P>z*** | **OR** | ***P>z*** | **OR** | ***P>z*** | **OR** | ***P>z*** | **OR** | ***P>z*** |
| **Rainfall variable** | | | | | | | | | | | | | | | | | |
| **Drought** | **No ®** |  |  |  |  |  |  |  |  |  |  |  |  |  |  |  |  |
|  | **Yes** | 1.69 | *<0.001* | 2.36 | *<0.001* | 1.52 | *<0.001* | 1.12 | *0.258* | 1.07 | *0.641* | 1.90 | *<0.001* | 1.81 | *<0.001* | 1.76 | *<0.001* |
| **Flood** | **No ®** |  |  |  |  |  |  |  |  |  |  |  |  |  |  |  |  |
|  | **Yes** | 0.98 | *0.603* | 0.98 | *0.728* | 0.93 | *0.168* | 1.11 | *0.119* | 0.88 | *0.129* | 0.96 | *0.451* | 0.85 | *0.031* | 1.60 | *<0.001* |
| **Household characteristics** | | | | | | | | | | | | | | | | | |
| **Household size** | **Up to 5 ®** |  |  |  |  |  |  |  |  |  |  |  |  |  |  |  |  |
|  | **More than 5** | 0.99 | *0.448* | 1.00 | *0.975* | 0.98 | *0.626* | 0.96 | *0.371* | 0.93 | *0.124* | 0.97 | *0.268* | 0.97 | *0.400* | 1.05 | *0.231* |
| **Household with out-migrant** | **Yes ®** |  |  |  |  |  |  |  |  |  |  |  |  |  |  |  |  |
|  | **No** | 1.47 | *<0.001* | 1.35 | *<0.001* | 1.64 | *<0.001* | 1.59 | *<0.001* | 1.57 | *<0.001* | 1.61 | *<0.001* | 1.43 | *<0.001* | 1.23 | *<0.001* |
| **Religion** | **Hinduism ®** |  |  |  |  |  |  |  |  |  |  |  |  |  |  |  |  |
|  | **Islam** | 1.13 | *<0.001* | 1.08 | *0.087* | 1.12 | *0.037* | 1.27 | *0.001* | 1.26 | *<0.001* | 1.01 | *0.870* | 0.89 | *0.640* | 1.74 | *0.076* |
|  | **Others** | 0.97 | *0.600* | 1.10 | *0.272* | 0.86 | *0.178* | 1.00 | *0.968* | 0.78 | *0.105* | 1.09 | *0.567* | 0.92 | *0.487* | 1.15 | *0.154* |
| **Social group** | **Others ®** |  |  |  |  |  |  |  |  |  |  |  |  |  |  |  |  |
|  | **Other backward castes** | 1.15 | *<0.001* | 1.18 | *<0.001* | 1.09 | *0.064* | 1.12 | *0.042* |  |  |  |  |  |  |  |  |
|  | **Scheduled castes** | 1.26 | *<0.001* | 1.29 | *<0.001* | 1.21 | *0.001* | 1.16 | *0.030* |  |  |  |  |  |  |  |  |
|  | **Scheduled tribes** | 1.44 | *<0.001* | 1.39 | *<0.001* | 1.51 | *<0.001* | 1.33 | *0.001* |  |  |  |  |  |  |  |  |
| **Land possession** | **Up to 1 hectare ®** |  |  |  |  |  |  |  |  |  |  |  |  |  |  |  |  |
|  | **1.01 to 4 hectares** | 0.93 | *0.014* | 1.04 | *0.337* | 0.82 | *<0.001* | 0.89 | *0.027* | 0.92 | *0.177* | 0.89 | *0.014* | 0.97 | *0.702* | 1.11 | *0.062* |
|  | **More than 4 hectares** | 0.66 | *<0.001* | 0.84 | *0.192* | 0.51 | *<0.001* | 0.65 | *0.001* | 0.73 | *0.053* | 0.54 | *<0.001* | 1.04 | *0.906* | 1.14 | *0.398* |
| **Occupation type** | **Self-employed in agriculture ®** |  |  |  |  |  |  |  |  |  |  |  |  |  |  |  |  |
|  | **Self-employed in non-agriculture** | 1.13 | *<0.001* | 1.16 | *0.002* | 1.10 | *0.069* | 1.10 | *0.134* | 0.92 | *0.216* | 1.16 | *0.002* | 1.19 | *0.025* | 1.10 | *0.315* |
|  | **Agricultural labour** | 1.61 | *<0.001* | 1.53 | *<0.001* | 1.64 | *<0.001* | 2.15 | *<0.001* | 1.44 | *<0.001* | 1.67 | *<0.001* | 1.40 | *<0.001* | 1.89 | *<0.001* |
|  | **Other labour** | 2.21 | *<0.001* | 2.46 | *<0.001* | 2.19 | *<0.001* | 1.83 | *<0.001* | 2.14 | *<0.001* | 2.05 | *<0.001* | 2.12 | *<0.001* | 2.59 | *<0.001* |
|  | **Others** | 0.92 | *0.031* | 0.99 | *0.887* | 0.91 | *0.232* | 0.82 | *0.001* | 1.09 | *0.261* | 0.84 | *0.012* | 0.94 | *0.575* | 0.76 | *0.001* |
| **MPCE tertiles** | **Lower ®** |  |  |  |  |  |  |  |  |  |  |  |  |  |  |  |  |
|  | **Middle** | 0.74 | *<0.001* |  |  |  |  |  |  | 0.69 | *<0.001* | 0.76 | *<0.001* | 0.74 | *<0.001* | 0.79 | *<0.001* |
|  | **Upper** | 0.63 | *<0.001* |  |  |  |  |  |  | 0.59 | *<0.001* | 0.67 | *<0.001* | 0.63 | *<0.001* | 0.66 | *<0.001* |
| **Individual characteristics** | | | | | | | | | | | | | | | | | |
| **Sex** | **Male ®** |  |  |  |  |  |  |  |  |  |  |  |  |  |  |  |  |
|  | **Female** | 0.11 | *<0.001* | 0.10 | *<0.001* | 0.09 | *<0.001* | 0.15 | *<0.001* | 0.05 | *<0.001* | 0.07 | *<0.001* | 0.11 | *<0.001* | 0.25 | *<0.001* |
| **Age** | **15-24 ®** |  |  |  |  |  |  |  |  |  |  |  |  |  |  |  |  |
|  | **25-34** | 1.15 | *<0.001* | 0.99 | *0.871* | 1.10 | *0.065* | 1.50 | *<0.001* | 1.35 | *<0.001* | 1.02 | *0.678* | 0.96 | *0.498* | 1.42 | *<0.001* |
|  | **35-44** | 0.78 | *<0.001* | 0.72 | *<0.001* | 0.74 | *<0.001* | 0.94 | *0.436* | 0.94 | *0.440* | 0.72 | *<0.001* | 0.67 | *<0.001* | 0.83 | *0.020* |
|  | **45-54** | 0.42 | *<0.001* | 0.35 | *<0.001* | 0.41 | *<0.001* | 0.69 | *<0.001* | 0.61 | *<0.001* | 0.35 | *<0.001* | 0.33 | *<0.001* | 0.52 | *<0.001* |
|  | **55-64** | 0.18 | *<0.001* | 0.14 | *<0.001* | 0.18 | *<0.001* | 0.26 | *<0.001* | 0.22 | *<0.001* | 0.15 | *<0.001* | 0.16 | *<0.001* | 0.21 | *<0.001* |
| **Marital status** | **Currently married ®** |  |  |  |  |  |  |  |  |  |  |  |  |  |  |  |  |
|  | **Never married** | 0.97 | *0.303* | 0.80 | *<0.001* | 0.89 | *0.026* | 1.53 | *<0.001* | 1.10 | *0.147* | 0.82 | *<0.001* | 0.73 | *<0.001* | 1.50 | *<0.001* |
|  | **Widowed** | 0.93 | *0.309* | 0.82 | *0.076* | 0.94 | *0.645* | 1.17 | *0.292* | 1.05 | *0.815* | 0.99 | *0.965* | 0.92 | *0.540* | 0.80 | *0.187* |
|  | **Divorced/separated** | 0.88 | *0.438* | 0.75 | *0.271* | 1.00 | *0.988* | 1.03 | *0.925* | 0.83 | *0.727* | 0.95 | *0.834* | 0.80 | *0.456* | 0.90 | *0.762* |
| **Educational level** | **Not literate ®** |  |  |  |  |  |  |  |  |  |  |  |  |  |  |  |  |
|  | **Literate but up to primary** | 0.98 | *0.491* | 1.03 | *0.394* | 0.97 | *0.489* | 0.96 | *0.505* | 1.19 | *0.006* | 1.05 | *0.250* | 1.02 | *0.620* | 0.73 | *<0.001* |
|  | **Above primary but up to secondary** | 0.75 | *<0.001* | 0.78 | *<0.001* | 0.82 | *<0.001* | 0.69 | *<0.001* | 0.89 | *0.102* | 0.81 | *<0.001* | 0.73 | *<0.001* | 0.57 | *<0.001* |
|  | **Above secondary** | 0.97 | *0.466* | 0.65 | *<0.001* | 0.92 | *0.209* | 1.26 | *0.002* | 1.08 | *0.370* | 0.74 | *<0.001* | 0.69 | *<0.001* | 1.76 | *<0.001* |
| **Geographical variable** | | | | | | | | | | | | | | | | | |
| **State** | **Bihar ®** |  |  |  |  |  |  |  |  |  |  |  |  |  |  |  |  |
|  | **Jammu and Kashmir** | 0.71 | *<0.001* | 0.47 | *0.001* | 0.55 | *<0.001* | 0.69 | *0.004* | 0.66 | *<0.001* | 0.58 | *0.001* | 0.63 | *0.042* | 0.67 | *0.568* |
|  | **Himachal Pradesh** | 0.29 | *<0.001* | 0.40 | *<0.001* | 0.27 | *<0.001* | 0.24 | *<0.001* | 0.20 | *<0.001* | 0.48 | *0.006* | 0.39 | *<0.001* | 0.35 | *0.004* |
|  | **Punjab** | 0.15 | *<0.001* | 0.19 | *<0.001* | 0.05 | *<0.001* | 0.18 | *<0.001* | 0.21 | *<0.001* | 0.15 | *<0.001* | 0.14 | *<0.001* |  |  |
|  | **Chandigarh** |  |  |  |  |  |  |  |  |  |  |  |  |  |  |  |  |
|  | **Uttaranchal** | 0.29 | *<0.001* | 0.26 | *<0.001* | 0.21 | *<0.001* | 0.35 | *<0.001* | 0.35 | *<0.001* | 0.20 | *<0.001* | 0.27 | *<0.001* |  |  |
|  | **Haryana** | 0.28 | *<0.001* | 0.29 | *<0.001* | 0.24 | *<0.001* | 0.26 | *<0.001* | 0.22 | *<0.001* | 0.22 | *<0.001* | 0.36 | *<0.001* |  |  |
|  | **Delhi** | 0.06 | *<0.001* |  |  |  |  | 0.05 | *<0.001* |  |  | 0.48 | *0.226* |  |  |  |  |
|  | **Rajasthan** | 0.56 | *<0.001* | 0.53 | *<0.001* | 0.55 | *<0.001* | 0.56 | *<0.001* | 0.50 | *<0.001* | 0.52 | *<0.001* | 0.77 | *0.010* | 0.77 | *0.311* |
|  | **Uttar Pradesh** | 0.78 | *<0.001* | 0.78 | *<0.001* | 0.75 | *<0.001* | 0.64 | *<0.001* | 0.65 | *<0.001* | 0.78 | *<0.001* | 0.85 | *0.023* | 1.18 | *0.641* |
|  | **Sikkim** | 0.08 | *<0.001* | 0.02 | *<0.001* | 0.04 | *<0.001* | 0.12 | *<0.001* | 0.02 | *<0.001* | 0.05 | *<0.001* | 0.05 | *0.003* | 0.24 | *<0.001* |
|  | **Arunachal Pradesh** | 0.44 | *<0.001* | 0.25 | *<0.001* | 0.39 | *<0.001* | 0.71 | *0.044* | 0.80 | *0.335* | 0.32 | *0.077* |  |  | 0.70 | *0.189* |
|  | **Nagaland** | 1.50 | *<0.001* | 0.53 | *0.549* | 1.15 | *0.580* | 1.27 | *0.104* | 1.65 | *0.382* | 0.43 | *0.258* |  |  | 2.15 | *0.004* |
|  | **Manipur** | 0.10 | *<0.001* | 0.22 | *<0.001* | 0.06 | *<0.001* | 0.09 | *<0.001* | 0.23 | *<0.001* | 0.07 | *<0.001* | 0.16 | *0.067* | 0.13 | *<0.001* |
|  | **Mizoram** | 0.18 | *<0.001* |  |  | 0.19 | *<0.001* | 0.20 | *<0.001* |  |  |  |  | 5.53 | *0.211* | 0.35 | *0.001* |
|  | **Tripura** | 0.38 | *<0.001* | 0.23 | *<0.001* | 0.32 | *<0.001* | 0.61 | *<0.001* | 0.43 | *<0.001* | 0.58 | *<0.001* | 0.39 | *<0.001* | 0.57 | *0.036* |
|  | **Meghalaya** | 0.29 | *<0.001* | 0.07 | *<0.001* | 0.28 | *<0.001* | 0.48 | *<0.001* | 0.85 | *0.493* | 0.17 | *0.024* |  |  | 0.47 | *0.007* |
|  | **Assam** | 0.52 | *<0.001* | 0.43 | *<0.001* | 0.52 | *<0.001* | 0.52 | *<0.001* | 0.43 | *<0.001* | 0.37 | *<0.001* | 0.60 | *0.021* | 1.44 | *0.158* |
|  | **West Bengal** | 0.80 | *<0.001* | 0.93 | *0.219* | 0.66 | *<0.001* | 0.56 | *<0.001* | 0.72 | *<0.001* | 0.69 | *0.002* | 0.78 | *0.002* | 1.58 | *0.072* |
|  | **Jharkhand** | 0.93 | *0.164* | 0.93 | *0.349* | 0.89 | *0.170* | 0.77 | *0.119* | 0.84 | *0.334* | 0.84 | *0.028* | 1.20 | *0.118* | 2.03 | *0.004* |
|  | **Orissa** | 0.44 | *<0.001* | 0.53 | *<0.001* | 0.34 | *<0.001* | 0.16 | *<0.001* | 0.22 | *<0.001* | 0.34 | *<0.001* | 0.59 | *<0.001* | 1.21 | *0.441* |
|  | **Chhattisgarh** | 0.57 | *<0.001* | 0.60 | *<0.001* | 0.50 | *<0.001* | 0.54 | *0.005* | 0.87 | *0.599* | 0.83 | *0.066* | 1.33 | *0.053* | 0.60 | *0.046* |
|  | **Madhya Pradesh** | 0.65 | *<0.001* | 0.61 | *<0.001* | 0.69 | *<0.001* | 0.58 | *<0.001* | 0.21 | *<0.001* | 0.59 | *<0.001* | 1.06 | *0.524* | 1.33 | *0.239* |
|  | **Gujarat** | 0.88 | *0.016* | 1.11 | *0.235* | 0.87 | *0.140* | 0.57 | *<0.001* | 0.40 | *<0.001* | 0.59 | *<0.001* | 0.52 | *<0.001* | 3.30 | *<0.001* |
|  | **Daman and Diu** | 0.26 | *<0.001* |  |  | 0.20 | *0.124* | 0.27 | *<0.001* |  |  | 0.49 | *0.025* |  |  | 0.33 | *0.142* |
|  | **Dadra and Nagar Haveli** | 0.01 | *<0.001* |  |  | 0.03 | *<0.001* |  |  |  |  |  |  |  |  | 0.04 | *0.002* |
|  | **Maharashtra** | 0.47 | *<0.001* | 0.63 | *<0.001* | 0.38 | *<0.001* | 0.29 | *<0.001* | 0.22 | *<0.001* | 0.53 | *<0.001* | 0.67 | *0.001* | 1.06 | *0.800* |
|  | **Andhra Pradesh** | 0.62 | *<0.001* | 0.85 | *0.010* | 0.49 | *<0.001* | 0.33 | *<0.001* | 0.33 | *<0.001* | 0.61 | *<0.001* | 0.78 | *0.006* | 1.68 | *0.043* |
|  | **Karnataka** | 0.70 | *<0.001* | 0.90 | *0.145* | 0.49 | *<0.001* | 0.53 | *<0.001* | 0.58 | *<0.001* | 0.68 | *<0.001* | 0.95 | *0.603* | 1.05 | *0.873* |
|  | **Goa** | 0.28 | *<0.001* | 0.14 | *0.059* | 0.25 | *0.003* | 0.30 | *0.005* | 0.18 | *<0.001* | 0.37 | *0.036* | 0.35 | *0.155* | 1.00 |  |
|  | **Lakshadweep** | 0.09 | *<0.001* |  |  | 0.05 | *0.004* | 0.14 | *0.001* |  |  |  |  |  |  | 0.14 | *0.002* |
|  | **Kerala** | 0.23 | *<0.001* | 0.14 | *<0.001* | 0.22 | *<0.001* | 0.28 | *<0.001* | 0.37 | *<0.001* | 0.21 | *<0.001* | 0.17 | *<0.001* | 0.17 | *0.018* |
|  | **Tamil Nadu** | 0.35 | *<0.001* | 0.18 | *<0.001* | 0.40 | *<0.001* | 0.68 | *0.012* | 0.95 | *0.862* | 0.31 | *<0.001* | 0.39 | *<0.001* | 0.38 | *0.099* |
|  | **Pondicherry** | 0.38 | *<0.001* | 0.07 | *<0.001* | 0.41 | *0.021* | 0.64 | *0.132* |  |  | 0.26 | *<0.001* | 0.68 | *0.218* |  |  |
|  | **Andaman and Nicobar** | 0.09 | *<0.001* | 0.32 | *0.274* | 0.03 | *<0.001* | 0.10 | *<0.001* | 0.02 | *<0.001* | 0.33 | *0.034* |  |  | 0.54 | *0.431* |
| **Constant** |  | 0.19 | *<0.001* | 0.22 | *<0.001* | 0.17 | *<0.001* | 0.09 | *<0.001* | 0.20 | *<0.001* | 0.26 | *<0.001* | 0.29 | *<0.001* | 0.08 | *<0.001* |
| **Number of obs** |  | 2,35,682 |  | 78,344 |  | 78,587 |  | 78,213 |  | 58,370 |  | 91,846 |  | 42,456 |  | 42,015 |  |
| **LR chi2(62)** |  | 20586.78 |  | 8461.11 |  | 6465.48 |  | 4401.53 |  | 4873.51 |  | 8894.16 |  | 4224.54 |  | 3735.94 |  |
| **Prob > chi2** |  | 0.00 |  | 0.00 |  | 0.00 |  | 0.00 |  | 0.00 |  | 0.00 |  | 0.00 |  | 0.00 |  |
| **Pseudo R2** |  | 0.18 |  | 0.17 |  | 0.18 |  | 0.17 |  | 0.22 |  | 0.20 |  | 0.17 |  | 0.18 |  |
| **Log likelihood** |  | -45723.34 |  | -20287.74 |  | -14271.52 |  | -10738.50 |  | -8537.71 |  | -17418.15 |  | -10092.00 |  | -8739.33 |  |

^a^OR denotes odds ratio.

® denotes the reference category.

**S11 Table. Description of variables.**

| **Variables** | **Measurements** | **Descriptions** |
| --- | --- | --- |
| **Dependent variable** | | |
| **Temporary migrant household** | 1=yes, 0=no | If a person is called a temporary migrant if he/she stayed away from village for 1 month or more but less than 6 months during last 365 days for employment or in search of employment. If at least one member of the household is classified as a temporary migrant that household is called a temporary migrant household. |
| **Rainfall variable** | | |
| **Drought** | 1=no, 2=yes | Districts which have experienced an annual rainfall negatively deviated by an amount of 40% or more than the normal annual rainfall (50 years average annual rainfall between 1951 to 2000 for that district) for at least one year between five years preceding the NSSO survey i.e. between 2003 to 2007 are classified as drought affected. The meteorological data used in this study is from IMD gridded rainfall binary file (0.25*0.25) and extracted with GRADS 2.2 and QGIS 3.10.0. |
| **Flood** | 1=no, 2=yes | The districts with a same amount of positive deviation of rainfall within the specified time period were classified as flood affected. Drought and flood data are merged with the NSSO data at district level. |
| **Household characteristics** | | |
| **Household size** | 1=up to 5, 2=more than 5 | A group of person normally living together and ordinarily taking food from a common kitchen constitute a household but normally left to the judgment of the head of the household. The number of members of a household is its size. |
| **Household with out-migrant** | 1=yes, 2=no | An out-migrant is any former member of the household who had migrated out any time in the past and is still alive to take up a residence in another village/town/district/state/country. |
| **Religion** | 1=Hinduism, 2=Islam, 3=Others | The religion of the head of the household |
| **Social group** | 1=Others, 2=OBC, 3=SC, 4=ST | The social group to which the head of the household belongs to. |
| **Land possession** | 1=up to 1 hectare, 2=1.01 to 4 hectares, 3=more than 4 hectares | Land possessed by the household within the country as on the date of survey is given by land owned (including land under ‘owner like possession’) + land leased in – land leased out + land held by the household but neither owned nor leased in (e.g., encroached land). |
| **Occupation type** | 1=self-employed in agriculture, 2=self-employed in non-agriculture, 3=agricultural labour, 4=other labour, 5=others | The economic activities which generates the main source of income for the household. |
| **MPCE tertiles** | 1=lower, 2=middle, 3=upper | Monthly per capita consumer expenditure in Rupees |
| **Household head characteristics** | | |
| **Sex** | 1=male, 2=female | Sex of the household head. Eunuch is recorded in code “1” |
| **Age** | 1=15-24, 2=25-34, 3=35-44, 4=45-54, 5=55-64, 6=up to 14, 7=65 and above | Age of the household head in completed years |
| **Marital status** | 1=currently married, 2=never married, 3=widowed, 4=divorced/separated | Marital status of the household head |
| **Educational level** | 1=not literate, 2=literate but up to primary, 3=above primary but up to secondary, 4=above secondary | The highest level of education successfully completed by the household head. A person who can both read and write a simple message with understanding in at least one language is literate. Those who are not able to do so, are not literate. |
| **Geographical variable** | | |
| **State** | 1=Bihar, 2=Jammu and Kashmir, 3=Himachal Pradesh, 4=Punjab, 5=Chandigarh, 6=Uttaranchal, 7=Haryana, 8=Delhi, 9=Rajasthan, 10=Uttar Pradesh, 11=Sikkim, 12=Arunachal Pradesh, 13=Nagaland, 14=Manipur, 15=Mizoram, 16=Tripura, 17=Meghalaya, 18=Assam, 19=West Bengal, 20=Jharkhand, 21=Orissa, 22=Chhattisgarh, 23=Madhya Pradesh, 24=Gujarat, 25=Daman and Diu, 26=Dadra and Nagar Haveli, 27=Maharashtra, 28=Andhra Pradesh, 29=Karnataka, 30=Goa, 31=Lakshadweep, 32=Kerala, 33=Tamil Nadu, 34=Pondicherry, 35=Andaman and Nicobar | State/ Union Territory of the sample household |

Source: Derived from NSSO 64th round (2007-2008).

**S12 Table. Characteristics of households in rural India (weighted), NSSO 64^th^ round (2007-2008) (in %).**

| **Variables** | **Total sample (N=** **159116743)** | **LEG (N=** **60226859)** | **MEG (N=** **54841817)** | **HEG (N=** **44048067)** | **Others (N=** **38310321)** | **OBC (N=** **68424722)** | **SC (N=** **34517260)** | **ST (N=** **17864443)** |
| --- | --- | --- | --- | --- | --- | --- | --- | --- |
| **Dependent variable** | | | | | | | | |
| **Temporary migrant** | | | | | | | | |
| No | 94.2 | 91.5 | 94.8 | 97.2 | 95.4 | 94.5 | 93.7 | 91.6 |
| Yes | 5.8 | 8.5 | 5.2 | 2.8 | 4.6 | 5.5 | 6.3 | 8.4 |
| **Rainfall variable** | | | | | | | | |
| **Drought** | | | | | | | | |
| No | 90.7 | 92.1 | 90.9 | 88.5 | 96.0 | 87.1 | 89.6 | 95.1 |
| Yes | 9.3 | 7.9 | 9.1 | 11.5 | 4.0 | 12.9 | 10.4 | 4.9 |
| **Flood** | | | | | | | | |
| No | 86.6 | 86.6 | 86.8 | 86.1 | 90.1 | 84.7 | 88.4 | 82.8 |
| Yes | 13.4 | 13.4 | 13.2 | 13.9 | 9.9 | 15.3 | 11.6 | 17.2 |
| **Household characteristics** | | | | | | | | |
| **Household size** | | | | | | | | |
| Up to 5 | 70.3 | 56.5 | 73.1 | 85.6 | 71.2 | 68.8 | 72.1 | 70.3 |
| More than 5 | 29.7 | 43.5 | 26.9 | 14.4 | 28.8 | 31.2 | 27.9 | 29.7 |
| **Household with out-migrant** | | | | | | | | |
| Yes | 30.4 | 24.9 | 30.1 | 38.2 | 35.2 | 31.6 | 28.2 | 19.7 |
| No | 69.6 | 75.1 | 69.9 | 61.8 | 64.8 | 68.4 | 71.8 | 80.3 |
| **Religion** | | | | | | | | |
| Hinduism | 84.9 | 85.8 | 85.4 | 82.9 | 69.0 | 88.2 | 93.6 | 89.0 |
| Islam | 10.3 | 11.3 | 10.8 | 8.3 | 24.1 | 10.0 | 0.5 | 0.7 |
| Others | 4.8 | 2.9 | 3.7 | 8.8 | 6.9 | 1.7 | 6.0 | 10.3 |
| **Social group** | | | | | | | | |
| Others | 24.1 | 16.1 | 23.2 | 36.0 |  |  |  |  |
| OBC | 43.0 | 41.1 | 45.7 | 42.3 |  |  |  |  |
| SC | 21.7 | 26.9 | 21.4 | 15.0 |  |  |  |  |
| ST | 11.2 | 15.9 | 9.8 | 6.6 |  |  |  |  |
| **Land possession** | | | | | | | | |
| Up to 1 hectare | 81.5 | 86.4 | 81.0 | 75.3 | 74.5 | 80.4 | 93.0 | 78.3 |
| 1.01 to 4 hectares | 16.5 | 12.7 | 17.3 | 20.8 | 21.8 | 17.4 | 6.8 | 20.6 |
| More than 4 hectares | 2.0 | 0.9 | 1.7 | 3.9 | 3.7 | 2.2 | 0.2 | 1.1 |
| **Occupation type** | | | | | | | | |
| Self-employed in agriculture | 34.9 | 28.9 | 37.7 | 39.7 | 43.0 | 38.0 | 17.7 | 39.5 |
| Self-employed in non-agriculture | 14.4 | 12.7 | 15.4 | 15.6 | 16.6 | 16.2 | 12.9 | 5.5 |
| Agricultural labour | 26.6 | 38.4 | 25.3 | 12.2 | 14.8 | 23.5 | 42.3 | 33.8 |
| Other labour | 11.5 | 12.5 | 11.8 | 10.0 | 8.8 | 10.5 | 16.8 | 11.0 |
| Others | 12.5 | 7.6 | 9.8 | 22.5 | 16.8 | 11.8 | 10.2 | 10.2 |
| **MPCE tertiles** | | | | | | | | |
| LEG | 37.9 |  |  |  | 25.4 | 36.2 | 46.9 | 53.6 |
| MEG | 34.5 |  |  |  | 33.2 | 36.6 | 34.0 | 30.0 |
| HEG | 27.7 |  |  |  | 41.4 | 27.3 | 19.1 | 16.3 |
| **Household head characteristics** | | | | | | | | |
| **Sex** | | | | | | | | |
| Male | 87.9 | 88.6 | 88.7 | 86.1 | 88.7 | 87.3 | 87.6 | 89.5 |
| Female | 12.1 | 11.4 | 11.3 | 13.9 | 11.3 | 12.7 | 12.4 | 10.5 |
| **Age** | | | | | | | | |
| 15-24 | 3.0 | 2.3 | 2.6 | 4.6 | 2.9 | 3.1 | 3.0 | 3.2 |
| 25-34 | 18.2 | 21.0 | 18.4 | 14.0 | 14.9 | 17.7 | 21.4 | 21.0 |
| 35-44 | 26.9 | 30.4 | 26.1 | 23.0 | 26.0 | 27.0 | 27.0 | 28.1 |
| 45-54 | 23.7 | 21.6 | 24.3 | 25.8 | 25.1 | 23.5 | 22.6 | 23.7 |
| 55-64 | 16.2 | 14.1 | 16.9 | 18.2 | 17.1 | 16.9 | 15.2 | 13.4 |
| Up to 14 | 0.9 | 1.1 | 0.6 | 1.0 | 0.5 | 0.4 | 0.6 | 3.9 |
| 65 and above | 11.1 | 9.6 | 11.1 | 13.2 | 13.5 | 11.4 | 10.1 | 6.6 |
| **Marital status** | | | | | | | | |
| Currently married | 84.8 | 86.9 | 86.2 | 80.1 | 85.4 | 85.0 | 84.8 | 82.5 |
| Never married | 3.2 | 2.0 | 2.1 | 6.1 | 3.2 | 2.8 | 2.4 | 5.8 |
| Widowed | 11.4 | 10.7 | 11.0 | 13.0 | 10.9 | 11.5 | 12.1 | 11.1 |
| Divorced/separated | 0.6 | 0.4 | 0.7 | 0.8 | 0.5 | 0.6 | 0.8 | 0.6 |
| **Educational level** | | | | | | | | |
| Not literate | 42.8 | 53.3 | 43.5 | 27.8 | 28.7 | 43.0 | 53.9 | 51.0 |
| Literate but up to primary | 28.2 | 28.5 | 29.2 | 26.4 | 29.3 | 28.3 | 25.2 | 30.9 |
| Above primary but up to secondary | 21.9 | 15.3 | 22.3 | 30.3 | 29.4 | 22.2 | 16.6 | 14.8 |
| Above secondary | 7.1 | 2.9 | 5.0 | 15.6 | 12.6 | 6.5 | 4.3 | 3.3 |
| **Geographical variable** | | | | | | | | |
| **State** | | | | | | | | |
| Bihar | 8.5 | 12.4 | 8.2 | 3.7 | 5.1 | 12.2 | 8.9 | 1.0 |
| Jammu and Kashmir | 0.8 | 0.2 | 1.0 | 1.5 | 2.4 | 0.2 | 0.6 | 0.1 |
| Himachal Pradesh | 0.9 | 0.2 | 0.7 | 1.9 | 2.1 | 0.2 | 1.0 | 0.5 |
| Punjab | 2.1 | 0.6 | 1.5 | 5.0 | 4.0 | 0.6 | 4.1 | 0.0 |
| Chandigarh | 0.0 | 0.0 | 0.0 | 0.1 | 0.1 | 0.0 | 0.0 | 0.0 |
| Uttaranchal | 0.9 | 0.4 | 1.0 | 1.4 | 2.0 | 0.3 | 0.8 | 0.4 |
| Haryana | 1.9 | 0.8 | 1.6 | 3.9 | 3.6 | 1.3 | 2.4 | 0.0 |
| Delhi | 0.1 | 0.0 | 0.0 | 0.4 | 0.2 | 0.0 | 0.3 | 0.0 |
| Rajasthan | 5.5 | 3.5 | 6.4 | 7.0 | 3.3 | 6.2 | 5.1 | 8.0 |
| Uttar Pradesh | 15.6 | 18.1 | 16.6 | 10.9 | 11.3 | 19.6 | 19.8 | 1.1 |
| Sikkim | 0.1 | 0.0 | 0.1 | 0.1 | 0.0 | 0.1 | 0.0 | 0.3 |
| Arunachal Pradesh | 0.1 | 0.1 | 0.1 | 0.2 | 0.1 | 0.0 | 0.0 | 0.7 |
| Nagaland | 0.1 | 0.0 | 0.0 | 0.3 | 0.0 | 0.0 | 0.0 | 0.8 |
| Manipur | 0.2 | 0.1 | 0.3 | 0.3 | 0.1 | 0.2 | 0.0 | 0.8 |
| Mizoram | 0.1 | 0.0 | 0.0 | 0.1 | 0.0 | 0.0 | 0.0 | 0.5 |
| Tripura | 0.4 | 0.4 | 0.4 | 0.5 | 0.4 | 0.2 | 0.4 | 1.3 |
| Meghalaya | 0.3 | 0.1 | 0.3 | 0.5 | 0.1 | 0.0 | 0.0 | 2.0 |
| Assam | 2.9 | 1.9 | 3.0 | 4.1 | 5.5 | 2.0 | 1.0 | 4.3 |
| West Bengal | 8.4 | 10.3 | 8.6 | 5.7 | 19.0 | 1.4 | 12.0 | 6.1 |
| Jharkhand | 2.6 | 3.3 | 2.9 | 1.3 | 1.0 | 2.6 | 1.9 | 7.5 |
| Orissa | 4.5 | 7.3 | 3.7 | 1.5 | 3.1 | 3.7 | 4.0 | 11.1 |
| Chhattisgarh | 2.5 | 3.6 | 2.4 | 0.9 | 0.4 | 2.2 | 1.4 | 9.6 |
| Madhya Pradesh | 5.8 | 8.2 | 5.1 | 3.5 | 3.5 | 5.5 | 4.7 | 13.9 |
| Gujarat | 4.1 | 2.3 | 4.1 | 6.6 | 3.5 | 4.5 | 2.2 | 7.9 |
| Daman and Diu | 0.0 | 0.0 | 0.0 | 0.0 | 0.0 | 0.0 | 0.0 | 0.0 |
| Dadra and Nagar Haveli | 0.0 | 0.0 | 0.0 | 0.0 | 0.0 | 0.0 | 0.0 | 0.1 |
| Maharashtra | 7.9 | 6.2 | 8.4 | 9.5 | 10.5 | 7.2 | 5.3 | 9.7 |
| Andhra Pradesh | 9.4 | 8.5 | 9.7 | 10.0 | 8.5 | 9.9 | 10.3 | 7.5 |
| Karnataka | 4.8 | 4.7 | 5.2 | 4.4 | 5.2 | 5.2 | 4.2 | 3.3 |
| Goa | 0.1 | 0.0 | 0.1 | 0.2 | 0.3 | 0.1 | 0.0 | 0.0 |
| Lakshadweep | 0.0 | 0.0 | 0.0 | 0.0 | 0.0 | 0.0 |  | 0.0 |
| Kerala | 3.4 | 1.2 | 2.3 | 8.0 | 4.2 | 4.6 | 1.8 | 0.8 |
| Tamil Nadu | 6.0 | 5.7 | 6.2 | 6.3 | 0.6 | 9.7 | 7.5 | 0.6 |
| Pondicherry | 0.1 | 0.0 | 0.1 | 0.1 | 0.0 | 0.1 | 0.1 |  |
| Andaman and Nicobar | 0.0 | 0.0 | 0.0 | 0.1 | 0.1 | 0.0 |  | 0.0 |

LEG: Lower economic group; MEG: Middle economic group; HEG: Higher economic group; OBC: Other Backward Class; SC: Scheduled Castes; ST: Scheduled Tribes.

**S13 Table. Temporary migration rates at household level by explanatory variables across different economic and social groups in rural India (weighted), NSSO 64^th^ round (2007-2008) (in %).**

| **Variables** | **Total sample** | **LEG** | **MEG** | **HEG** | **Others** | **OBC** | **SC** | **ST** |
| --- | --- | --- | --- | --- | --- | --- | --- | --- |
| **Rainfall variable** | | | | | | | | |
| **Drought** | | | | | | | | |
| No | 5.9 | 8.7 | 5.3 | 2.8 | 4.7 | 5.8 | 6.4 | 8.1 |
| Yes | 4.6 | 6.0 | 4.7 | 3.1 | 1.9 | 3.8 | 5.2 | 14.3 |
| **Flood** | | | | | | | | |
| No | 5.7 | 8.3 | 5.2 | 2.7 | 4.9 | 5.6 | 6.4 | 6.8 |
| Yes | 6.6 | 9.9 | 5.6 | 3.4 | 2.6 | 5.4 | 5.8 | 16.3 |
| **Household characteristics** | | | | | | | | |
| **Household size** | | | | | | | | |
| Up to 5 | 4.7 | 7.1 | 4.6 | 2.6 | 3.6 | 4.5 | 5.4 | 6.6 |
| More than 5 | 8.5 | 10.4 | 6.9 | 3.9 | 7.3 | 7.9 | 8.7 | 12.8 |
| **Household with out-migrant** | | | | | | | | |
| Yes | 5.4 | 8.8 | 5.1 | 2.6 | 4.2 | 4.8 | 6.3 | 11.2 |
| No | 6.0 | 8.4 | 5.3 | 2.9 | 4.9 | 5.9 | 6.3 | 7.8 |
| **Religion** | | | | | | | | |
| Hinduism | 5.6 | 8.2 | 5.1 | 2.7 | 3.4 | 5.4 | 6.5 | 8.6 |
| Islam | 8.4 | 11.5 | 7.3 | 4.2 | 9.3 | 7.2 | 10.2 | 5.4 |
| Others | 3.3 | 6.3 | 2.8 | 2.3 | 1.2 | 3.6 | 2.6 | 7.1 |
| **Social group** | | | | | | | | |
| Others | 4.6 | 8.4 | 4.7 | 2.3 |  |  |  |  |
| OBC | 5.5 | 7.9 | 5.2 | 2.8 |  |  |  |  |
| SC | 6.3 | 8.3 | 5.0 | 3.6 |  |  |  |  |
| ST | 8.4 | 10.6 | 7.1 | 3.8 |  |  |  |  |
| **Land possession** | | | | | | | | |
| Up to 1 hectare | 6.1 | 8.7 | 5.5 | 2.8 | 5.2 | 5.9 | 6.3 | 8.6 |
| 1.01 to 4 hectares | 4.7 | 7.5 | 4.2 | 2.8 | 3.2 | 4.3 | 6.6 | 8.0 |
| More than 4 hectares | 3.0 | 7.4 | 2.8 | 1.7 | 2.3 | 2.8 | 10.9 | 5.9 |
| **Occupation type** | | | | | | | | |
| Self-employed in agriculture | 4.9 | 7.7 | 4.5 | 2.6 | 3.7 | 4.7 | 6.2 | 7.1 |
| Self-employed in non-agriculture | 5.1 | 7.4 | 4.8 | 2.8 | 3.7 | 5.6 | 5.9 | 5.0 |
| Agricultural labour | 7.6 | 9.4 | 6.2 | 3.9 | 7.8 | 7.6 | 6.7 | 9.9 |
| Other labour | 9.3 | 12.6 | 8.7 | 4.6 | 9.8 | 7.7 | 8.5 | 16.8 |
| Others | 2.1 | 2.7 | 2.4 | 1.8 | 2.4 | 2.1 | 1.9 | 1.9 |
| **MPCE tertiles** | | | | | | | | |
| LEG | 8.5 |  |  |  | 8.4 | 7.9 | 8.3 | 10.6 |
| MEG | 5.2 |  |  |  | 4.7 | 5.2 | 5.0 | 7.1 |
| HEG | 2.8 |  |  |  | 2.3 | 2.8 | 3.6 | 3.8 |
| **Household head characteristics** | | | | | | | | |
| **Sex** | | | | | | | | |
| Male | 6.1 | 9.0 | 5.5 | 2.9 | 4.8 | 5.9 | 6.7 | 8.8 |
| Female | 3.4 | 4.8 | 3.1 | 2.2 | 2.9 | 3.2 | 3.4 | 5.6 |
| **Age** | | | | | | | | |
| 15-24 | 6.6 | 9.1 | 6.3 | 5.1 | 6.8 | 4.8 | 9.1 | 8.1 |
| 25-34 | 6.3 | 8.2 | 5.6 | 3.5 | 4.9 | 6.0 | 6.5 | 8.9 |
| 35-44 | 5.9 | 8.6 | 4.8 | 2.4 | 4.6 | 5.5 | 6.7 | 8.4 |
| 45-54 | 6.7 | 9.9 | 6.4 | 3.4 | 5.5 | 6.3 | 7.2 | 9.9 |
| 55-64 | 5.7 | 9.5 | 5.1 | 2.4 | 4.5 | 5.5 | 6.1 | 9.2 |
| Up to 14 | 0.0 | 0.0 | 0.0 | 0.0 | 0.0 | 0.0 | 0.0 | 0.0 |
| 65 and above | 3.4 | 5.2 | 3.4 | 1.5 | 2.6 | 3.8 | 3.0 | 5.5 |
| **Marital status** | | | | | | | | |
| Currently married | 6.1 | 8.9 | 5.5 | 2.7 | 4.8 | 5.7 | 6.7 | 9.1 |
| Never married | 3.4 | 3.2 | 2.2 | 4.1 | 4.4 | 3.7 | 4.1 | 1.3 |
| Widowed | 4.5 | 6.7 | 4.0 | 2.6 | 3.1 | 4.6 | 4.5 | 7.3 |
| Divorced/separated | 2.4 | 5.1 | 2.0 | 1.0 | 2.6 | 2.0 | 3.4 | 1.1 |
| **Educational level** | | | | | | | | |
| Not literate | 7.2 | 9.4 | 6.0 | 3.5 | 6.5 | 6.8 | 6.8 | 9.9 |
| Literate but up to primary | 5.8 | 8.5 | 4.9 | 3.2 | 5.0 | 5.5 | 6.6 | 7.5 |
| Above primary but up to secondary | 4.0 | 6.2 | 4.5 | 2.1 | 3.2 | 3.9 | 4.9 | 6.5 |
| Above secondary | 3.0 | 5.3 | 4.0 | 2.1 | 2.9 | 2.9 | 3.4 | 3.9 |
| **Geographical variable** | | | | | | | | |
| **State** | | | | | | | | |
| Bihar | 13.4 | 16.0 | 11.6 | 6.1 | 10.9 | 14.0 | 13.6 | 10.1 |
| Jammu and Kashmir | 6.7 | 12.7 | 8.1 | 4.8 | 6.7 | 3.4 | 8.1 | 21.1 |
| Himachal Pradesh | 2.1 | 3.9 | 2.6 | 1.5 | 1.6 | 3.0 | 2.5 | 3.2 |
| Punjab | 1.3 | 5.6 | 0.1 | 1.0 | 0.8 | 1.3 | 1.8 | 0.0 |
| Chandigarh | 0.0 | 0.0 | 0.0 | 0.0 | 0.0 | 0.0 | 0.0 | 0.0 |
| Uttaranchal | 1.8 | 1.9 | 1.9 | 1.7 | 2.3 | 1.4 | 1.4 | 0.0 |
| Haryana | 1.5 | 2.7 | 1.9 | 0.9 | 1.2 | 1.8 | 1.6 | 0.0 |
| Delhi | 0.6 | 0.0 | 0.0 | 0.7 | 0.0 | 4.2 | 0.0 | 0.0 |
| Rajasthan | 5.9 | 9.8 | 5.0 | 4.1 | 3.7 | 4.8 | 8.3 | 8.0 |
| Uttar Pradesh | 6.2 | 7.5 | 5.9 | 3.8 | 5.3 | 5.9 | 7.2 | 8.1 |
| Sikkim | 1.3 | 1.8 | 1.1 | 1.3 | 0.1 | 1.0 | 0.1 | 2.0 |
| Arunachal Pradesh | 7.0 | 5.1 | 5.5 | 9.3 | 7.7 | 1.2 | 0.0 | 7.0 |
| Nagaland | 12.9 | 4.2 | 11.5 | 13.1 | 16.8 | 6.9 | 0.0 | 13.0 |
| Manipur | 1.4 | 1.6 | 0.6 | 2.4 | 6.1 | 0.8 | 6.5 | 1.0 |
| Mizoram | 1.7 | 0.0 | 2.9 | 1.4 | 0.0 | 0.0 | 4.4 | 1.7 |
| Tripura | 1.1 | 1.1 | 1.1 | 1.1 | 1.4 | 1.4 | 0.8 | 0.9 |
| Meghalaya | 5.3 | 3.3 | 5.3 | 5.6 | 16.6 | 10.7 | 0.0 | 4.1 |
| Assam | 5.2 | 6.8 | 5.9 | 3.7 | 6.5 | 3.2 | 5.5 | 5.2 |
| West Bengal | 8.5 | 12.5 | 6.2 | 3.1 | 9.4 | 6.9 | 6.5 | 11.7 |
| Jharkhand | 10.4 | 12.8 | 10.0 | 2.7 | 6.0 | 10.5 | 16.5 | 8.5 |
| Orissa | 4.3 | 5.0 | 3.7 | 1.4 | 4.0 | 3.0 | 4.4 | 6.0 |
| Chhattisgarh | 4.3 | 5.9 | 2.5 | 1.9 | 7.6 | 4.2 | 7.6 | 3.2 |
| Madhya Pradesh | 8.4 | 11.0 | 7.1 | 2.6 | 2.1 | 5.8 | 9.9 | 14.8 |
| Gujarat | 7.4 | 14.2 | 7.9 | 3.8 | 1.5 | 5.1 | 1.7 | 21.1 |
| Daman and Diu | 3.5 | 0.0 | 9.2 | 3.0 | 0.0 | 8.8 | 0.0 | 5.5 |
| Dadra and Nagar Haveli | 0.1 | 0.0 | 0.2 | 0.0 | 0.0 | 0.0 | 0.0 | 0.1 |
| Maharashtra | 2.9 | 4.5 | 2.5 | 2.0 | 2.0 | 3.0 | 3.4 | 4.4 |
| Andhra Pradesh | 3.3 | 4.4 | 3.6 | 1.5 | 1.0 | 3.5 | 3.9 | 5.9 |
| Karnataka | 3.2 | 5.2 | 1.9 | 1.9 | 3.0 | 2.5 | 4.6 | 3.6 |
| Goa | 3.6 | 3.8 | 7.9 | 0.5 | 0.6 | 12.3 | 2.6 | 0.0 |
| Lakshadweep | 1.7 | 0.0 | 3.4 | 1.4 | 0.0 | 0.0 |  | 1.7 |
| Kerala | 1.9 | 2.0 | 2.6 | 1.6 | 1.3 | 2.3 | 1.2 | 3.0 |
| Tamil Nadu | 3.3 | 2.8 | 3.3 | 4.0 | 2.1 | 3.1 | 4.1 | 0.9 |
| Pondicherry | 3.4 | 0.1 | 2.3 | 5.4 | 0.0 | 3.3 | 3.6 |  |
| Andaman and Nicobar | 2.3 | 1.8 | 1.3 | 2.8 | 0.6 | 8.1 |  | 3.5 |

LEG: Lower economic group; MEG: Middle economic group; HEG: Higher economic group; OBC: Other Backward Class; SC: Scheduled Castes; ST: Scheduled Tribes.
